# Retrogressive Thaw Slumps Produce a Changing Disturbance Regime for Arctic Stream Invertebrates

**DOI:** 10.1111/gcb.70701

**Published:** 2026-03-09

**Authors:** Maria Dolan, Jordan Musetta‐Lambert, Krista S. Chin, Steven V. Kokelj, Suzanne E. Tank, Jennifer Lento, Michael Power, Joseph M. Culp

**Affiliations:** ^1^ Wilfrid Laurier University Waterloo Canada; ^2^ Environment and Climate Change Saskatoon Canada; ^3^ Government of the Northwest Territories Yellowknife Canada; ^4^ University of Alberta Edmonton Canada; ^5^ University of New Brunswick Fredericton Canada; ^6^ University of Waterloo Waterloo Canada

**Keywords:** Arctic freshwater biodiversity, arctic streams, benthic macroinvertebrates, climate warming, disturbance regime, long‐term monitoring, permafrost thaw, thaw slumps

## Abstract

For many Arctic rivers and streams, climate‐driven intensification of permafrost thaw slumping is a major source of disturbance to aquatic habitats. Thaw slumps are dynamic landforms that severely increase total suspended solids (TSS) and nutrients in downstream reaches and can persist over decades. Effects may differ in magnitude as slumps cycle through periods of higher and lower activity, with expansion of retrogressive slumps increasing over time. Increases in TSS are known to cause reduced invertebrate abundance and diversity in impacted watersheds; however, it remains unclear if water quality and critical aquatic biodiversity have recovered after prolonged exposure to slumps. Here, we examined decadal‐scale effects of slumps and environmental change on benthic macroinvertebrates (BMI) by comparing environmental and BMI data collected between 2010–2014 and a recent sampling campaign from 2021. High TSS and nutrient concentrations observed during 2010–2014 persisted in slump‐impacted sites in 2021, with no significant change in TSS and total nutrient concentrations after the 10‐year exposure period. TSS continued to act as a nonspecific stressor on BMI, as abundance remained significantly lower in impacted streams compared to reference streams. Although total abundance within reference and impacted sites did not differ significantly between sampling periods, abundance and richness of disturbance tolerant taxa was greater in 2021 as compared to 2010–2014 across all sites, with differences linked to lower precipitation in 2021. These community compositional changes were reflected in increased Shannon‐Weiner diversity between sampling campaigns. Overall, the number of thaw slumps upstream was an important driver of both BMI abundance and diversity across sampling periods and will likely continue to be an important determinant of benthic macroinvertebrate communities as the number and size of thaw slumps continues to increase across the circumpolar Arctic.

## Graphical abstract

Climate change is causing permafrost to thaw, resulting in the formation of large craters in the ground called thaw slumps, which deposit sediments and nutrients into rivers and continually impact stream ecosystems over time. We returned to slump impacted streams 10 years after an initial sample period and found that slumps permanently change streams by increasing sediment concentrations, which negatively impacts the insects that live at the bottom of the stream. The number of aquatic insects cannot recover, and insect communities are becoming dominated by groups that typically indicate long‐term disturbance.

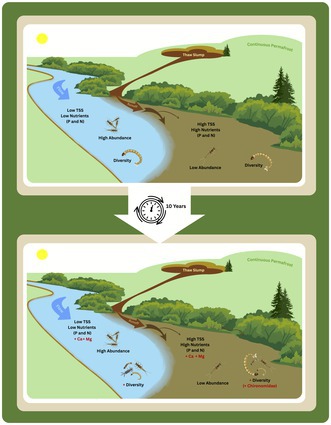

## 1 Introduction

**FIGURE 1:**
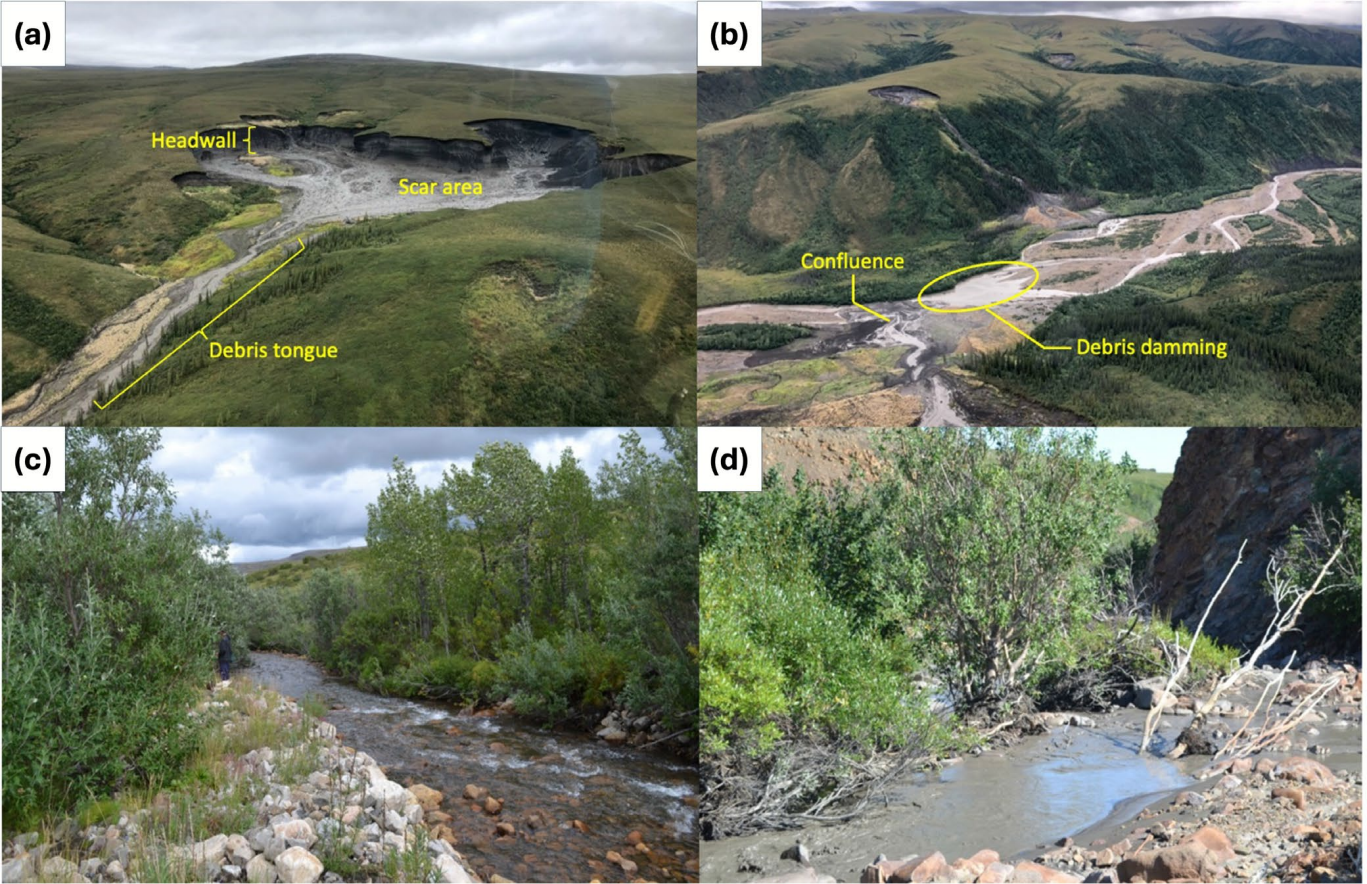
Retrogressive thaw slump morphology and stream impact. (a) A retrogressive thaw demonstrating an ice rich headwall between 30 m (shortest point) and 50 m (tallest point) in height, and a debris tongue of mud slurry flowing downhill. In September 2018, the slump was 980 m long, and 630 m at the widest section, with a total area of approximately 221, 600m^2^. (b) An example of direct thaw slump debris entering into a river system at a confluence, and a resulting “debris dam” impeding streamflow. (c) A typical reference stream in the Peel Plateau, clear and unimpacted by slump inputs. (d) A typical slump impacted stream in the peel plateau, demonstrating high turbidity and sediment. All photographs by Maria Dolan.

Climate change is rapidly altering Arctic freshwater environments, with increasing temperatures and changing precipitation patterns greatly affecting rivers and their watersheds (Nilsson et al. 2015). Permafrost thaw in the form of retrogressive thaw slumps (RTS) is a major driver of change in Arctic river ecosystems (Kokelj et al. 2021). RTS are chronic landslides that form in ice‐rich terrain because of thaw‐driven erosional processes induced by extreme summer temperatures (Lewkowicz and Way 2018), high rainfall (Lacelle et al. 2010; Kokelj et al. 2015), or coastal erosion (Lantuit and Pollard 2008). The erosion of soil exposes a headwall of ground ice that ablates upslope as thawing materials accumulate in a scar area (Figure 1; Nesterova et al. 2024). When the scar area is saturated, thawed materials can flow downslope to form a debris tongue, transporting sediment, solutes, and nutrients from slopes to valley bottoms (Kokelj et al. 2013, 2015). RTS are dynamic landforms that typically initiate as small landslides or slumps and enlarge over time, varying in size and activity across watersheds. In general, a warming and wetter climate has amplified the chronic nature of thaw slumps (Segal et al. 2016) where disturbances progressively enlarge, escalating the duration and volume of thawed materials conveyed to downslope. As such, thaw slumps are increasing in number and size across ice‐rich permafrost landscapes in the circumpolar Arctic. They are most intense and dynamic in glaciated regions where permafrost has preserved relict ground ice (Kokelj et al. 2017). Areas of greatest RTS abundance include the Western Canadian Arctic (Lacelle et al. 2015; Lewkowicz and Way 2018), the Alaskan and Brooks mountain ranges (Balser et al. 2014), Siberia (Biskaborn et al. 2013; Séjourné et al. 2015), and the Tibetan Plateau (Luo et al. 2019). Some of the most pronounced effects caused by RTS occur in rivers and streams, where a single thaw slump has the potential to displace hundreds of thousands of cubic meters of sediment into adjacent watercourses (Kokelj et al. 2021; van der Sluijs et al. 2023), increasing surface water turbidity, solutes, and total suspended solids (TSS) in reaches downstream of slump confluences (Kokelj et al. 2013). Mass wasting can alter hydrological flow (i.e., through impoundment of water by debris tongues), cause severe restructuring of stream habitat and geomorphology (Kokelj et al. 2021), and change the ecological function of fluvial ecosystems (Levenstein et al. 2018; Chin et al. 2016; Vonk et al. 2015). Therefore, the physical and stoichiometric effects of slumps can have significant negative impacts on riverine biota and ecological processes (Chin et al. 2016; Shakil et al. 2020).

The timing and magnitude of suspended sediments and debris delivered to streams by RTS may represent a changing disturbance regime in impacted fluvial ecosystems, having both acute and chronic stress effects. The sudden thaw‐driven intensification in slope sediment flux represents an acute stressor that has immediate and intense impacts on stream ecosystems. Shot‐lasting but intensive stress from thaw‐driven sediment flux is cyclical in nature due to the diurnal correlation between slump activity and solar insolation, and varies with large rain events (Kokelj et al. 2015). Acute stress effects can significantly alter stream habitat and biotic communities through sediment deposition that increases substrate embeddedness and channel scouring due to sediment saltation. Further, sedimentation negatively affects all light dependent groups (i.e., epilithic algae and macrophytes; Levenstein et al. 2018; Jones, Collins, et al. 2012; Jones, Murphy, et al. 2012), as suspended sediments can cause decreases in light available for photosynthesis. Thaw slumps also represent a chronic stress that unfolds over decadal time scales, continually affecting streams. For example, debris tongues and their deposits store slump material within or directly adjacent to the stream, building reserves of sediment up to 26.2 ha in area (Kokelj et al. 2015). Such deposits will take decades to be cleared by flowing water, and will persist after the associated slump becomes inactive. Through the presence of debris tongues and growth of sediment deposits, slumps can continually modify river channel morphology by changing flow patterns and stream velocity (Kokelj et al. 2015), affecting sediment transport and deposition, and ultimately resulting in a new, defining, disturbance regime (Kokelj et al. 2021). Slumps and their deposits may introduce meltwater and associated solutes into river environments, influencing river thermal regimes and consistently altering water chemistry parameters, such as pH, dissolved oxygen, and nutrient concentrations over decades (Kokelj et al. 2013; Chin et al. 2016; Kendrick et al. 2018). Furthermore, nitrogen (N), phosphorus (P), and carbon (C) previously stored within permafrost or leached from the surrounding land may be released to the stream and assimilated by organisms in the stream food web, ultimately decreasing the productivity of stream ecosystems (Kendrick et al. 2018; O'Donnell et al. 2020; Shakil et al. 2020). While these changes in the fluvial environment can produce long‐term effects on the structural and functional properties of stream biological assemblages, the temporal scale and the direction and magnitude of exposure to the chronic and acute stress effects of slumps remain unclear.

Although most research on the responses of benthic macroinvertebrates (BMI) to permafrost thaw has focussed on lakes (e.g., Moquin et al. 2014; Cohen et al. 2021), previous studies in fluvial systems have examined the effects of thaw slumps on stream biota by comparing BMI assemblages of unimpacted streams to those found in sites impacted by varying degrees of slumping (Chin et al. 2016; Levenstein et al. 2021). In regions dominated by ice‐rich tills, such as the Peel Plateau, Northwest Territories, Canada, thawing soils generally comprise very fine‐grained materials (30%–60% clay, 40%–70% fine silt). When mobilized by mass wasting processes, such fine‐grained material can cause high concentrations of TSS in river systems and result in low BMI abundance, diversity, and richness (Jones, Collins, et al. 2012; Jones, Murphy, et al. 2012; Chin et al. 2016; Mustonen et al. 2016; Levenstein et al. 2021). At low to moderate levels of sedimentation, the creation of additional habitat types may benefit some benthic taxa, initially increasing diversity (Lenat et al. 1981; Wlodarska‐Kowalczuk et al. 2005). However, the extreme increase in sediment load downstream of slumps fills interstitial spaces between rocks that provide valuable habitat for most BMI groups, overriding any initial potential beneficial effect of sedimentation, such as increased habitat heterogeneity (Lemly 1982; Larsen and Ormerod 2010; Chin et al. 2016). Increased suspended sediments can damage delicate invertebrate‐feeding apparatuses, especially in filter‐feeding taxa (Bilotta and Brazier 2008; Jones, Collins, et al. 2012; Jones, Murphy, et al. 2012). Further, many invertebrate taxa rely on algae as a food source, so decreases in algal biomass caused by increased turbidity (Levenstein et al. 2018) have the potential to affect grazer taxa (Kendrick et al. 2018). Finally, greater amounts of sedimentation will also increase the drift out of affected habitats, especially in drift‐prone taxa (Culp et al. 1986; Levenstein et al. 2021). While BMI responses to the effects of slumps have been studied (e.g., Chin et al. 2016; Levenstein et al. 2021), the age of slumps and duration of impact exposure in previous studies were unknown. Therefore, current knowledge may not reflect any long‐term temporal variability of BMI in response to extended exposure to permafrost thaw cycles. For example, it is unclear whether invertebrate communities may undergo additional restructuring in response to changes in slump activity over decadal periods.

Previous research has provided valuable insights into how permafrost thaw affects biological stream health parameters such as BMI abundance and community structure (Chin et al. 2016; Kendrick et al. 2018; O'Donnell et al. 2020; Levenstein et al. 2021), and benthic algal biomass and decomposition rates (Mustonen et al. 2016; Levenstein et al. 2018). In their spatially extensive study of the Peel Plateau region, Chin et al. (2016) observed a significant decline in invertebrate abundance along a gradient of increasing suspended sediments from thaw slumps. However, little to no long‐term research has been done to determine how persistent intensifying thaw slumps will continue to affect stream biota. As climate change continues to drive increases in temperatures and changes to precipitation in the Arctic, thaw slump activity is likely to increase in frequency and magnitude (Segal et al. 2016), and more information is needed on BMI responses to the effects of long‐term slump activity. Thus, it is important to return to previously sampled sites to determine if reported patterns (i.e., Chin et al. 2016) remain, or if BMI assemblages have changed due to either the intensification of slump activity or acclimation of stream biota to disturbance conditions.

Here, we examine the impacts of thaw slumps on freshwater streams by determining the stream reach and catchment scale drivers of BMI community change over two periods separated by 10 years. To accomplish this, we: (1) classify thaw slump impact level at sites to reflect current knowledge of slump activity, (2) determine how stream habitat and water quality in 2021 have changed relative to a baseline period (2010–2014), (3) describe how BMI communities differ between sampling periods, and (4) investigate the correlation between temporal changes in BMI and landscape features. Between 2010 and 2021, accelerating slump activity across the watershed was expected to have increased the amount of suspended sediment in previously studied sites (Chin et al. 2016; Levenstein et al. 2021). We hypothesized that years of intensifying instream total suspended solids (TSS), sediment transport, and deposition from RTS have a cumulative impact on BMI abundance and diversity. Thus, we predicted that the chronic input of slump materials over the 10 years between sampling events will have led to lower BMI abundance and diversity.

## 2 Materials and Methods

### 2.1 Study Area

**FIGURE 2:**
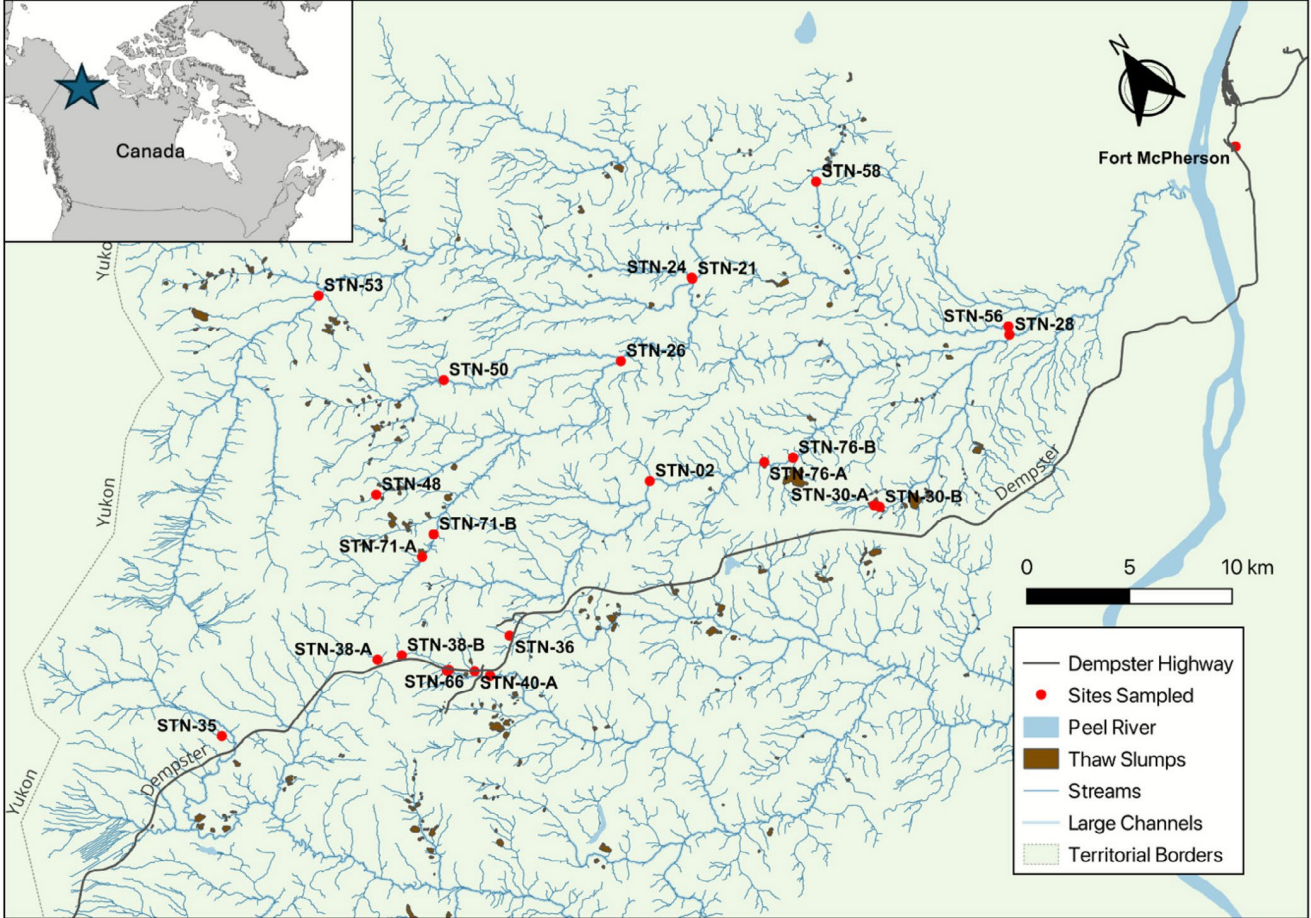
Site map of Stony Creek Watershed in the Peel Plateau, Northwest Territories, Canada. Inset, upper left, denotes geographic position of the sampling sites relative to northern Canada. Map lines delineate study areas and do not necessarily depict accepted national boundaries.

The Peel Plateau region (~24,000 km^2^) is located along the eastern edge of the Richardson and Mackenzie mountains in traditional Gwich'in lands, Northwestern Canada (Figure 2). The area marks the western boundary of the former Laurentide Ice Sheet, which shaped the landscape via its advance and retreat, contributing large stores of Pleistocene relict ground ice that is now preserved in continuous permafrost (Kokelj et al. 2017). The climate of the Peel Plateau is characterized by long, cold winters (average winter air temperature −22.5°C) and short, cool summers (average summer air temperature 10°C). Between 2010 and 2020, the mean annual air temperature was −5.1°C, and mean total annual rainfall was 358.6 mm (Kokelj et al. 2022). While rainfall has been increasing since 1986 when climate monitoring in the region began, the highest total annual rainfall from the 2010 to 2021 period was recorded in 2014 at 467.4 mm, and the lowest was recorded in 2021 at 68.1 mm (Kokelj et al. 2022). The land is dominated by hummock‐filled tundra at higher elevations (> 470 m asl), boreal forest at lower elevations (< 330 m asl) and in sheltered river valleys, and shrub tundra in the transition zone between the two (O'Neill et al. 2015; Kokelj et al. 2017). Stream networks drain eastward into the Peel River (Teetł'it Gwinjik), where typical plateau streams are small and pristine, with gravel‐cobble beds (Kokelj et al. 2013). The upper portions often originate in unglaciated mountainous terrain and are largely free of significant thermokarst disturbances (Kokelj et al. 2021). However, the fluvially‐incised nature of the streams and rivers and the ice‐rich landscape form favorable conditions for the development of thaw slumps throughout the plateau below elevations of ~700 m, with approximately 580 thaw slumps having been identified in the area to date (van der Sluijs et al. 2023), and a density of approximately 1 slump per 41 km^2^. Here, we focus on streams in the Stony Creek (Gwatoh Taii Njik) catchment (~1100 km^2^), and Vittrekwa Creek (Vitreekwaa Viteetshìk) headwaters, located on the southern portion of the Peel Plateau, around 30 km southwest of Fort McPherson (Teetł'it Zheh; Figure 2).

### 2.2 Site Selection and Classification

In August 2021, a total of 24 stream sites in the Stony Creek and Vittrekwa River watersheds were selected from those previously studied by Chin et al. (2016) and Levenstein et al. (2021) between 2010 and 2014 (Table 1, Table S1). Selected sites included a mixture of low to high‐order streams impacted by varying degrees of slump activity, with stream size generally < 15 m in wetted width due to wading constraints. Each site was sampled for substrate composition, water chemistry, and BMI communities.

**TABLE 1:** List of sites sampled and associated stream characteristics for the first time a site was sampled in 2010–2014, and the 2021 sampling campaigns.

						2010–2014				2021			
Site	Latitude	Longitude	Slump class	Number of upstream slumps	△TSS	TSS (mg/L)	Wetted width (m)	Average depth (cm)	Velocity (m/s)	TSS (mg/L)	Wetted width (m)	Average depth (cm)	Velocity (m/s)
STN‐02	67.261	−135.53119	2	9	−34	42	7.2	18.34	0.83	8	7.2	6.4	0.615
STN‐21	67.348776	−135.48769	4	58	3738^a^	332	12.3	37.6	1.01	4070	—	40	—
STN‐24	67.34929	−135.4884	2	54	8180^a^	340	15.1	32.2	0.76	8520	—	23	—
STN‐26	67.312569	−135.56701	3	25	−168^a^	370	10.8	20.9	0.48	202	11.3	27.96	1.062
STN‐28	67.327045	−135.12543	3	65	593^a^	342	14.1	23.9	0.53	935	10.4	21.5	0.611
STN‐30‐A	67.251697	−135.26969	1	2	−44	48	1.3	11.67	0.43	4	0.24	1.9	0.198
STN‐30‐B	67.252476	−135.27611	4	3	35660^a^	3440	1.9	10.5	0.25	39,100	1.1	—	—
STN‐35	67.146347	−136.00868	1	0	17	3	10.5	16.7	0.6	20	—	6.54	0.435
STN‐36	67.192977	−135.68625	1	0	21	3	13.5	14	0.35	24	2.43	3.77	0.316
STN‐38‐A	67.181095	−135.83498	1	0	12.5	1.5	1.9	10.5	0.212	14	0.76	6.83	0.454
STN‐38‐B	67.183294	−135.80765	3	1	101874^a^	126	2.75	7	0.39	102,000	0.8	1.8	0.235
STN‐40‐A	67.175442	−135.70727	4	7	28492^a^	8	0.6	21	0.261	28,500	7.46	6.86	0.708
STN‐40‐B	67.177198	−135.72475	3	1	4722^a^	558	2.3	7.67	0.36	5280	0.81	6.43	0.444
STN‐48	67.252421	−135.84125	1	1	2	4	4.45	15.67	0.1	6	8.2	8.5	0.843
STN‐50	67.302619	−135.76793	3	30	1110^a^	5460	4.1	15	0.5	6570	8.3	17.5	1.058
STN‐53	67.342023	−135.90439	3	42	200^a^	4880	17.2	19.3	0.48	5080	—	12.3	0.92
STN‐56	67.330561	−135.12678	3	146	2476^a^	794	15.15	16.4	0.42	3270	—	28.3	0.871
STN‐58	67.391776	−135.34863	3	12	−348^a^	364	1.9	7	0.54	16	2.5	3.83	0.919
STN‐66	67.177419	−135.75373	3	2	−298^a^	382	1	14.3	0.3	84	1.1	4.27	0.556
STN‐67	67.177134	−135.7563	2	1	12	8	2.2	13	0.3	20	2.26	4.2	0.236
STN‐71‐A	67.22598	−135.78746	1	0	0	8	5	17	0.372	8	7.9	10.7	0.836
STN‐71‐B	67.23592	−135.77501	3	1	616^a^	224	7.6	13.4	0.471	840	4	10.96	1.002
STN‐76‐A	67.27017	−135.40164	3	17	−5	8	5.1	—	—	3	11	12.52	0.203
STN‐76‐B	67.27239	−135.36906	3	18	−260^a^	266	6.1	23.4	0.31	6	4.5	14.08	0.439

*Note:* Slump class 1 = no active slumps upstream; slump class 2 = active upstream slumps < 1 ha in active area AND > 1.5 km away from the site; slump class 3 = active upstream slumps > 1 ha in active area OR < 1.5 km away from the site; slump class 4 = active upstream slumps > 1 ha in active area AND < 1.5 km away from the site; TSS, total suspended solids; ΔTSS, sites where change in TSS > 100 mg/L. Slump class is based on RTS conditions described in van der Sluijs et al. (2023).

^a^ ΔTSS, sites where change in TSS > 100 mg/L.

### 2.3 Stream Habitat and Water Chemistry

Substrate composition was measured using the Wolman pebble count method following protocols described by Environment and Climate Change Canada (Environment Canada 2012). For this procedure, 100 substrate particles were randomly selected from the stream bed and diameter was measured along the intermediate axis to compute the Wolman D_50_ index. Substrate embeddedness (≈25%, 50%, 75%) was also noted for 10% of the samples. On‐site point water quality measurements were conducted with a ProDSS Multiparameter Digital Water Quality Meter (YSI). Measurements included: temperature (°C), pH, specific conductance (μS/cm), and dissolved oxygen (% and mg/L). Water samples were collected and tested for nutrients (total phosphorus and total nitrogen), major ions (Ca, Mg and Na), turbidity, metals (total and dissolved), and TSS (mg/L) at the National Laboratory for Environmental Testing (NLET), Environment Canada, Burlington, following protocols described in Environment and Climate Change Canada [ECCC] (2020).

### 2.4 Invertebrate Collection and Processing

BMI were sampled at different sites in 2010, 2012, 2013, 2014, and 2021 (see Table S1 for years of sampling for each site) following Chin et al. (2016) using a standardized 3‐min traveling kick, with a triangle frame and 400 μm mesh kick net cleaned between each site (standardized Canadian Aquatic Biomonitoring Network (CABIN) protocol; Environment Canada 2012). Multiple microhabitats (e.g., large boulders, riffles and runs) were covered to capture natural variation in invertebrate communities across dominant habitat types. After 3 min, the net was rinsed to collect all biological material, which was further preserved in 70% ethanol before transport to the laboratory for benthic invertebrate identification. Samples were sorted to genus and species level where possible using ECCC's CABIN protocol (Environment Canada 2012). Subsampling was conducted with the use of a Marchant box to a minimum of 300 organisms, or 50 cells. The percent subsampled from the total sample was recorded, and invertebrate counts were corrected to represent 100% of the total sample where necessary. Invertebrate sample sorting and identification were conducted by taxonomists certified by the Society of Freshwater Science (Cordillera Consulting).

### 2.5 Slump Physical Attribute Analysis

Physical and geomorphological slump metrics were used to describe landscape attributes of the slumps and potential drivers of instream ecological change. Slump metrics were extracted from a data repository created by the Government of the Northwest Territories Geological Survey Permafrost Mapping Collective using aerial surveys and Landsat landscape change detection protocols for the period between 2011 and 2017 using QGIS (van der Sluijs et al. 2023). Selected metrics included the number of thaw slumps upstream from the sample site, proximity of the site to the confluence of the nearest active slump (m), the area of the nearest active slump (m^2^), the nearest slump activity (% cover of saturated scar area measured by visual inspection), and the total active area of the nearest slump (area of slump × slump activity; m^2^) (van der Sluijs et al. 2023).

To reflect current knowledge of physical thaw slump attributes, all sites were categorized into four slump impact classes describing the impact intensity from the nearest active slump on the site. Slump class was determined using the active area of the slump and the distance of the slump to the nearest sample site. Active area was used to represent the potential for runoff into the stream by each slump, and distance to the nearest slump was used as it was previously found to be an important determinant of invertebrate abundance (Chin et al. 2016). Class 1 sites contained unimpacted sites that did not have active slumps upstream (*n* = 6); Class 2 sites included minimally impacted sites, where the active upstream slumps had an active area of less than 1 ha and were over 1.5 km away from the site (*n* = 3); Class 3 sites contained moderately impacted sites, where active upstream slumps either had an active area greater than 1 ha or were less than 1.5 km from the site (*n* = 12); Class 4 sites included highly impacted sites, where the active upstream slumps had an active area greater than 1 ha and were located less than 1.5 km from the site (*n* = 3; Table 1).

### 2.6 Statistical Methods

To assess whether changes in water chemistry or invertebrate assemblages occurred between sampling periods as a result of slumps, analysis was completed by separating sites into categories by sampling year (i.e., 2010–2014 or 2021), and slump class (i.e., 1, 2, 3, and 4). As sites were initially sampled at irregular intervals during the ice‐free season between 2010 and 2014 (Table S1), a “past” category was created using results from the first year a site was sampled, allowing for detailed changes in variables across site classifications to be revealed through a side‐by‐side comparison of two sampling periods: past (2010–2014) vs. present (2021).

Standard BMI metrics including total abundance, Ephemeroptera (E), Plecoptera (P), and Trichoptera (T) abundance, chironomid abundance, richness, EPT richness, chironomid richness, evenness, and Shannon‐Weiner Diversity (SWD) were calculated from assemblage data at each site using the lowest possible taxonomic resolution. SWD was used as it highlights species richness and evenness, making it ideal for sensing community changes over periods of time. EPT metrics represented taxa sensitive to thaw disturbances, while the Dipteran family Chironomidae was used in representing “tolerant” taxa metrics to facilitate univariate analysis. Chironomids were classified as “tolerant” to slump disturbance as they are known to dominate streams with high degrees of sedimentation (Armitage et al. 1983; Raunio et al. 2007; Molineri et al. 2020). EPT family and Chironomid subfamily were then used in multivariate analysis of BMI community assemblages to highlight the diversity within EPT and Chironomidae and determine which specific families and subfamilies are driving any change in community composition between sampling periods.

Two‐way ANOVA and Tukey's post hoc honestly significant difference (HSD) tests were run for each BMI metric and physical environment parameter (conductivity, pH, turbidity, Wolman D50, TN, total phosphorus, Mg, Ca and Na concentrations) to test for any significant differences in means among the different thaw slump impact classes and sampling periods. Because of the established linkage between abundance and TSS (see Chin et al. 2016), an additional ANOVA was completed to determine if abundance differed between sampling periods at sites that had large temporal differences in TSS (i.e., a change in TSS of over 100 mg/L between sampling periods). Variables were log‐transformed where appropriate to meet test assumptions of normality and homogeneity of variance. Most variables met normality assumptions, apart from log_10_ TSS, log_10_ total nitrogen (TN), and chironomid richness. However, nonparametric analysis provided results consistent with those of the parametric test for TSS, which confirmed that ANOVA was not affected by deviations from normality.

Permutational multivariate analysis of variance (PERMANOVA) and nonmetric multidimensional scaling (NMDS) ordination were used to compare BMI metrics and community composition among slump classes, sampling periods, and the interaction between slump class and sampling period. For analysis of BMI metrics, sites were described by a total of 14 variables, including: %Ephemeroptera, %Plecoptera, %Trichoptera, %Chironomidae, total taxonomic richness, taxonomic richness for Ephemeroptera, Plecoptera, Trichoptera, and Chironomidae, total log_10_ abundance, log_10_ abundance for Chironomidae and EPT, SWD, and evenness. Analysis of BMI community composition occurred at the family and subfamily level (Table S2). Rare taxa (present at less than 20% of sites) were removed to eliminate any misleading impact they might have on the analysis (Arscott et al. 2006), resulting in 22 different invertebrate families and chironomid subfamilies. Relative abundance was calculated for each of the resulting taxa to control for variation in total abundance among sites. Similarity percentage (SIMPER) was used to determine which BMI metrics and taxa were contributing to any observed differences highlighted by PERMANOVA. Significance tests for PERMANOVA and SIMPER were based on 999 permutations of transformed data, and all multivariate analyses were based on Bray–Curtis similarity matrices.

Simple linear regression analyses were completed for both log_10_ total abundance and SWD using data from all years to determine important drivers of invertebrate communities regardless of sampling period. Abundance and SWD were selected as response variables because abundance is known to have a strong association with slump impact (Chin et al. 2016; Levenstein et al. 2021), and SWD is representative of trends in both evenness and richness (Strong 2016). Variables used as predictors were those previously shown to impact BMI metrics, including: log_10_ TSS (mg/L) and distance to nearest active slump (m); and newly obtained measurements of slump impacts, including: number of upstream slumps and active area of the nearest active slump (%). Year was also used as a predictor to account for other dynamic environmental parameters (i.e., water depth, wetted width, velocity, and rainfall) that may have affected abundance or diversity. As rainfall differed significantly across sampling campaigns, invertebrate variables with year as a significant predictor also considered total yearly rainfall as a possible independent variable to determine if hydroclimate influenced the observed differences across sampling years.

Predictors that explained a significant amount of variation in abundance and diversity in simple linear regressions were used to create a list of candidate multiple regression models, and the Akaike information criterion corrected for small sample sizes (AICc) was used to select a model to represent trends in invertebrate abundance and diversity (Symonds and Moussalli 2011). Models within 2 AICc units of the “best” model were used to assess the importance of individual parameters, and Akaike weights for each model that contained the parameter of interest were summed to obtain weighted importance (∑*W*_i_). The Variance Inflation Factor (VIF) was used to confirm collinearity assumptions for linear models were met. Finally, Analysis of covariance (ANCOVA) was used to determine if the slopes of significant relationships were the same across sampling periods by testing the interaction between significant predictor variables and sampling period. All statistical analyses were completed using RStudio (version 4.3.0; R Core Team 2024) with the *ggplot2* (Wickham 2016), *vegan* (version 2.6.8; Oksanen et al. 2024), *dplyr* (Wickham et al. 2023) and *car* (Fox and Weisberg 2019) packages on data from Dolan (2026). Significance was judged at *α* = 0.05.

## 3 Results

### 3.1 Water Chemistry

**FIGURE 3:**
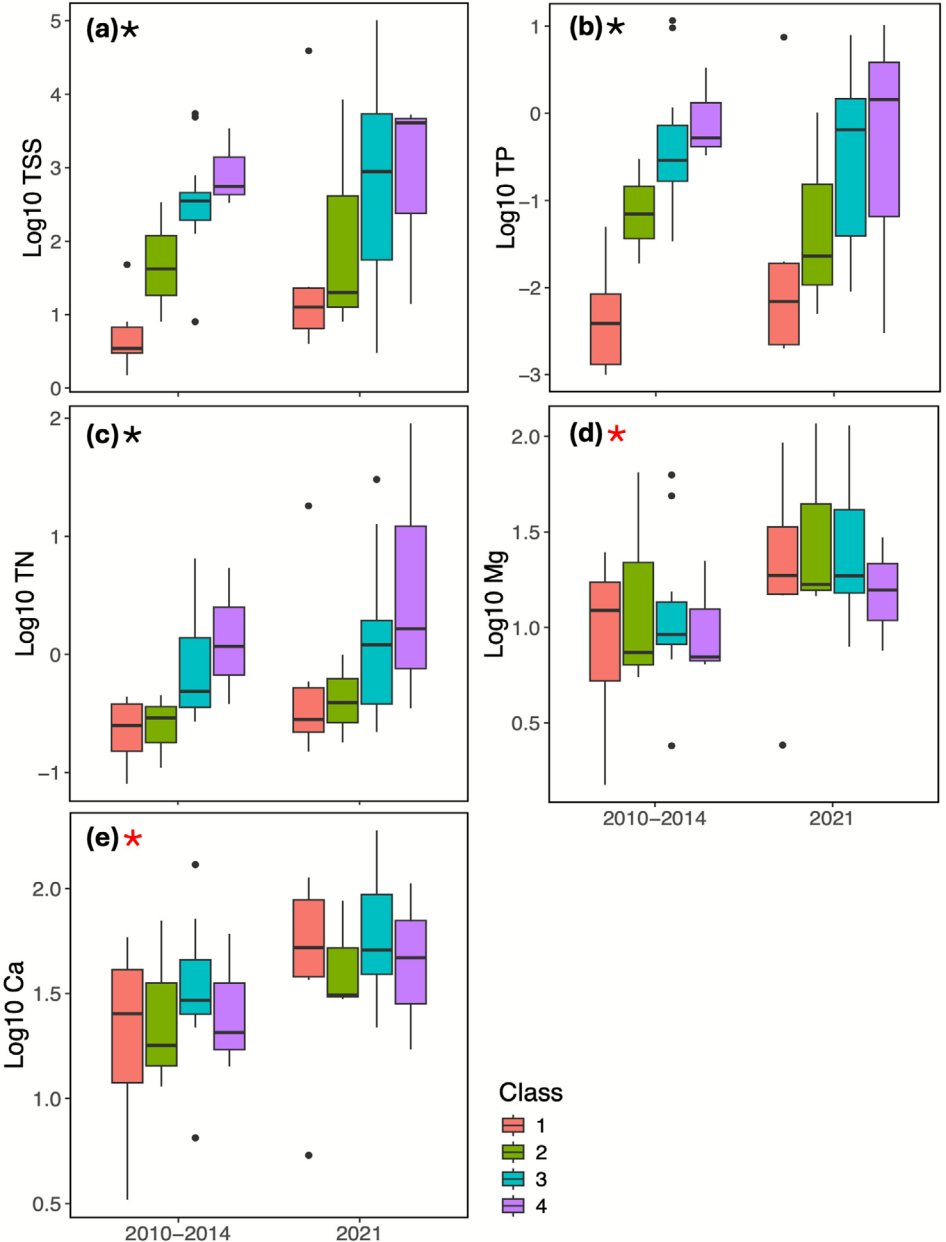
Box plots for water chemistry variables with a significant difference among sampling periods or slump impact classifications, including (a) log_10_ TSS, (b) log_10_ TP, (c) log_10_ TN, (d) log_10_ Magnesium and (e) log_10_ Calcium. Boxes represent the interquartile range, with the center line is the median. Plots with a black star represent metrics that differ significantly between slump classes, and plots with a red star represent metrics that differ significantly between sampling periods. TSS, total suspended solids; TP, total phosphorous; TN, total nitrogen.

There were significant differences in TSS (*F*_(3,40)_ = 4.869, *p* = 0.006) and nutrient concentrations (log_10_ TP *F*_(3,40)_ = 7.938, *p* < 0.001; log_10_ TN *F*_(3,40)_ = 3.517, *p* = 0.026) among sites impacted by thaw slumps compared to unimpacted sites. Unimpacted sites (Class 1) had significantly lower concentrations of TSS (Figure 3a), TP (Figure 3b), and TN (Figure 3c) than moderately impacted sites (Class 3) (log_10_ TSS *p* = 0.001; log_10_ TP *p* < 0.001; log_10_ TN *p* = 0.015) and highly impacted sites (Class 4) (log_10_ TSS *p* = 0.004; log_10_ TP *p* < 0.001; log_10_ TN *p* = 0.025). Although TSS, TP, and TN concentrations differed among slump classes, mean values did not differ significantly between sampling periods. However, greater variation in TSS was detected in 2021 at minimally impacted sites (Class 2: 2010–14 coefficient of variation (CV) = 48%; 2021 CV = 80%), moderately impacted sites (Class 3: 2010–14 CV = 37%; 2021 CV = 52%), and highly impacted sites (Class 4: 2010–14 CV = 18%; 2021 CV = 52%). Of the 24 sites, 45.8% showed TSS increases greater than 100 mg/L, 16.7% showed TSS decreases greater than 100 mg/L, and 37.5% changed less than this amount (Table 1). Despite mean TSS and nutrient concentrations not differing significantly between sampling periods, dissolved magnesium (Figure 3d) and calcium (Figure 3e) were significantly higher on average in 2021 than 2010–14 (log_10_ Mg *F*_(1,40)_ = 7.134, *p* = 0.011; log_10_ Ca *F*_(1,40)_ = 7.122, *p* = 0.011). Dissolved cation concentrations did not differ by slump class. There were no significant differences (*p* > 0.053) detected in pH, conductivity, or Wolman D50 among slump impact classes or between sampling periods, and no significant differences in the interaction between slump classification and sampling periods for all water chemistry ANOVAs (Table S3).

### 3.2 Univariate Invertebrate Metrics

**FIGURE 4:**
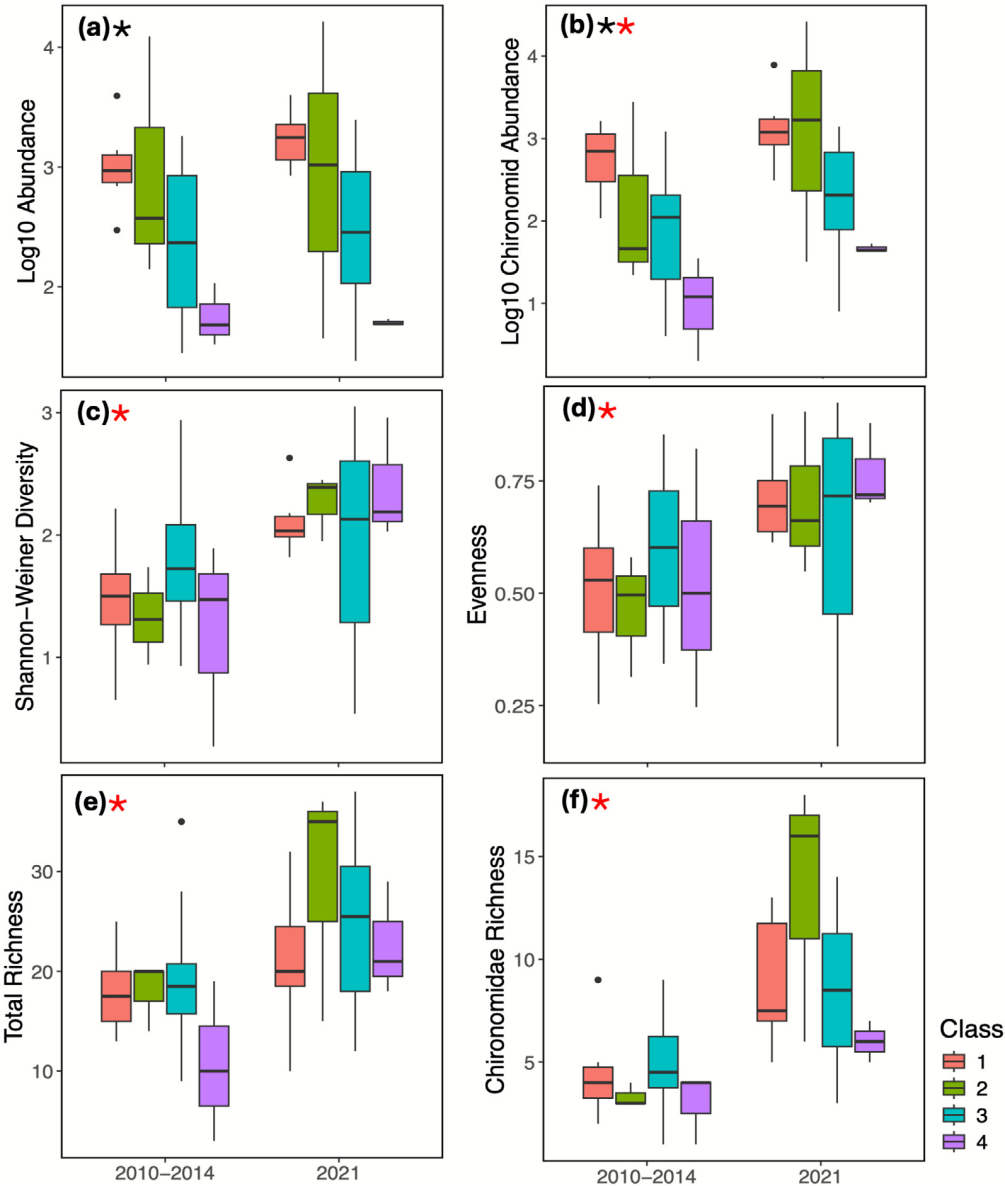
Boxplots for invertebrate metrics with a significant difference among sampling years or slump impact classifications, including (a) log_10_ total invertebrate abundance, (b) log_10_ Chironomidae abundance, (c) Shannon‐Weiner diversity index, (d) Evenness, (e) Total richness, and (f) Chironomidae richness. Boxes represent the interquartile range, with the center line at the median. Plots with a black star represent metrics that differ significantly between slump classes, and plots with a red star represent metrics that differ significantly between sampling periods. No plots had significant interaction effects between year and slump impact.

Total abundance (*F*_(3,40)_ = 8.048, *p* < 0.001), Chironomidae abundance (*F*_(3,40)_ = 7.614, *p* < 0.001), and EPT abundance (*F*_(3,40)_ = 4.996, *p* = 0.005) differed significantly between slump classes, whereas a significant difference between sampling periods (*F*_(1,40)_ = 5.818, *p* = 0.021) was only found for chironomid abundance. For both sampling campaigns, sites in highly impacted classes had significantly lower total abundance (Figure 4a), Chironomidae abundance (Figure 4b), and EPT abundance (*p* = 0.002). Total (*p* = 0.001), chironomid (*p* = 0.002), and EPT abundance (*p* = 0.002) in Class 1 (unimpacted) sites were higher on average than Class 4 (highly impacted) sites. Total abundance (*p* = 0.033) and Chironomidae abundance (*p* = 0.019) were also significantly higher on average in Class 1 as compared to Class 3 (moderately impacted) sites. Further, Chironomidae abundance (*p* = 0.018) and EPT abundance (*p* = 0.028) were significantly higher on average in Class 2 (minimally impacted) sites than in Class 4 sites. While total abundance (*F*_(1,40)_ = 0.246, *p* = 0.622) and EPT abundance (*F*_(1,40)_ = 0.421, *p* = 0.520) for all classes grouped together did not differ significantly between sampling periods, there were more Chironomidae individuals on average in 2021 as compared to 2010–2014 (Figure 4b). Sites that demonstrated large changes in TSS (i.e., > 100 mg/L) between sampling campaigns did not show any corresponding significant differences (*F*_(2,40)_ = 1.341, *p* = 0.272) in mean abundances (Figure S1). Sites with no TSS change and sites that decreased in TSS generally showed higher mean abundances in 2021, although differences were not significant (*p* = 0.776). There was also no significant difference (*p* = 0.978) in mean abundance in sites that increased in TSS concentration between sampling periods (Figure S1).

Univariate comparisons of SWD (Figure 4c), evenness (Figure 4d), and taxonomic richness (Figure 4e) revealed strong differences among sampling periods but not slump impact classes. SWD (*F*_(1,40)_ = 9.423, *p* = 0.003), taxonomic richness (*F*_(1,40)_ = 8.071, *p* = 0.010), and evenness (*F*_(1,40)_ = 5.291, *p* = 0.03) were significantly higher on average in 2021 than in 2010–2014. EPT richness did not differ between sampling years (*F*_(1,40)_ = 0.420, *p* = 0.521) or slump impact classes (*F*_(3,40)_ = 2.115, *p* = 0.113), but chironomid richness was higher on average in 2021 than 2010–2014 (*F*_(1,40)_ = 25.107, *p* < 0.001) (Figure 4f). Moreover, metrics related to diversity did not differ significantly (*p* > 0.968) among slump classifications, and the interaction between slump classification and sampling period was not significant (*F*_(1,40)_ = 1.274, *p* = 0.296; Table S4).

### 3.3 Multivariate Approaches to Metrics and Community Composition

**FIGURE 5:**
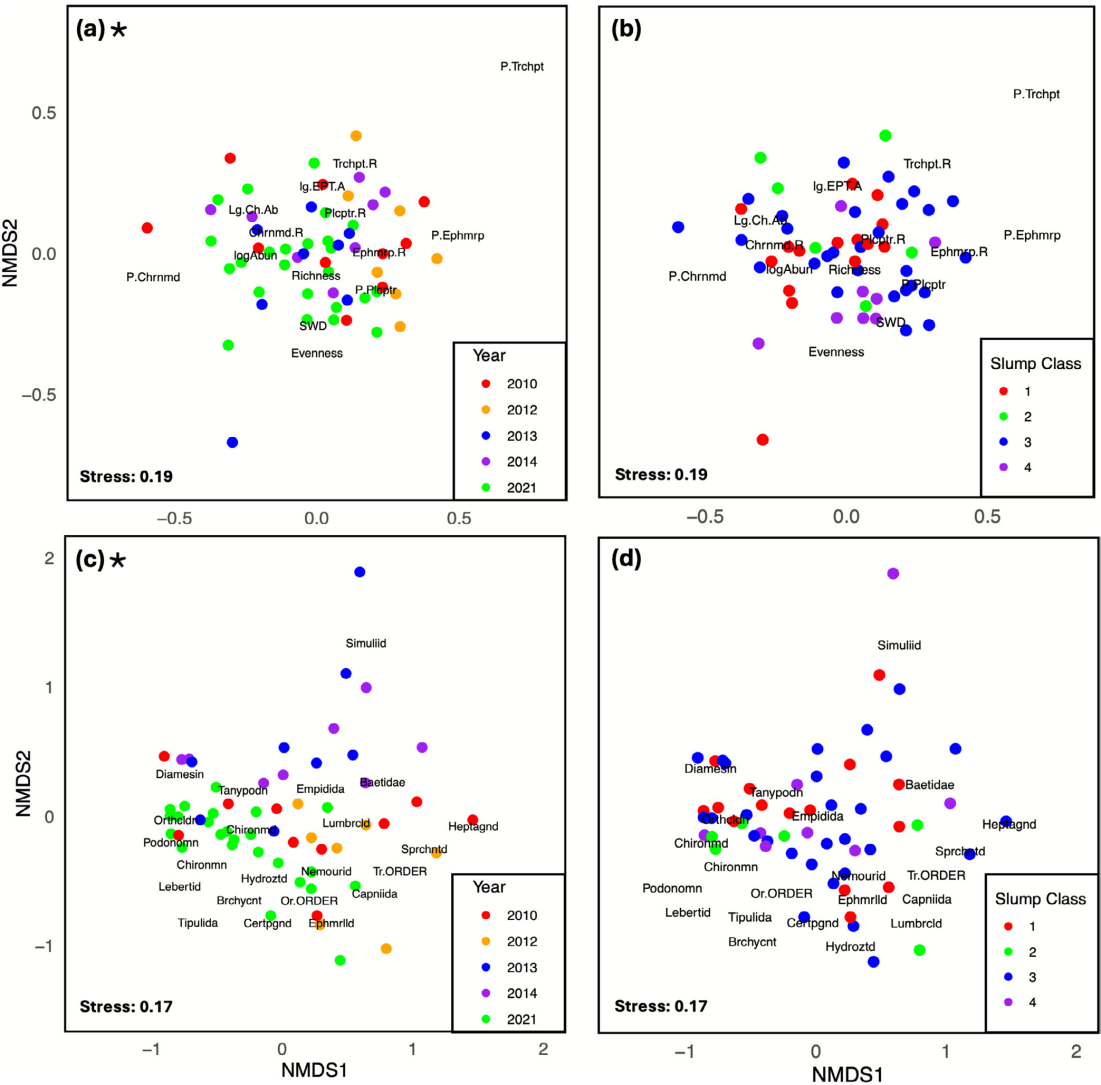
Nonmetric multidimensional scaling (NMDS) for invertebrate data. Panels (a) and (b) show sites summarized by invertebrate metrics, and panels (c) and (d) show sites summarized by relative abundance sommunity composition. Color depicts year sampled (panels a and c) and site class (panels b and d). Plots with a black star represent significant corresponding PERMANOVA tests. Plots (a and b) abbreviations: Chrnmd.R, Chironomidae richness; Ephmrp.R, Ephemeroptera richness; Lg.Ch.Ab, log chirononomidae abundance; lg.EPT.A, logEPT abundance; logAbun, log total abundance; P.Chrnmd, percent Chironomidae; P.Ephmrp, percent Ephemeroptera; P.Plcptr = percent Plecoptera; P.Trchpt, percent Trichoptera; Plcptr.R, Plecoptera richness; Trchpt.R, Trichoptera richness. Plots (c and d) abbreviations: Brchycnt, Brachycentridae; Capniida, Capniidae; Certpgnd, Ceratopogonidae; Chironmd, Chironomidae; Chironmn, Chironominae; Diamesin, Diamesinae; Empidida, Empididae; Ephmrlld, Ephemerellidae; Heptagnd, Heptageniidae; Hydrozid, Hydroziidae; Lebertid, Lebertiidae; Lumbrcld, Lumbriculiidae; Or.ORDER, Oribitida; Orthcldn, Orthocladiinae; Podonomn, Podonominae; Simuliid, Simuliidae; Sprchntd, Spherconidae; Tanypodn, Tanypodinae; Tipulida, Tipuliidae; Tr.ORDER, Trombidiformes.

BMI metrics and community composition were more similar within sampling period (Figure 5a,c) than within slump classification (Figure 5b,d). PERMANOVA showed a significant difference between sampling periods for BMI metrics (*F*_(1,40)_ = 10.706, *p* < 0.001; Table S5) and community composition (*F*_(1,40)_ = 5.159, *p* < 0.001; Table S6). When summarized by invertebrate metrics, SIMPER revealed chironomid richness and total richness contributed most consistently to the dissimilarity between sampling periods, where sites sampled in 2021 had greater total richness and chironomid richness compared to all years in the 2010–2014 period (Table S7). When sites were summarized by community composition, SIMPER revealed that Chironomidae and the subfamily Orthocladiinae contributed most consistently to the dissimilarity between 2021 and 2010–2014 (Table S8). PERMANOVA showed no significant differences between sites grouped by slump class while summarized by either BMI metrics (*F*_(3,40)_ = 1.140, *p* = 0.328) or community composition (*F*_(3,40)_ = 0.790, *p* = 0.730).

### 3.4 Explanatory Models to Describe Invertebrate Variation Over Time

**FIGURE 6:**
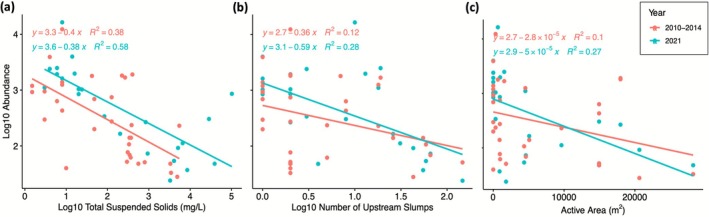
Linear regressions of log_10_ Abundance with significant slump predictor variables divided by sampling campaign. (a) Depicts log_10_ abundance regressed against log_10_ TSS, (b) depicts log_10_ abundance regressed against log_10_ number of upstream slumps, and (c) depicts log_10_ abundance regressed against slump active area. TSS, total suspended solids.

**FIGURE 7:**
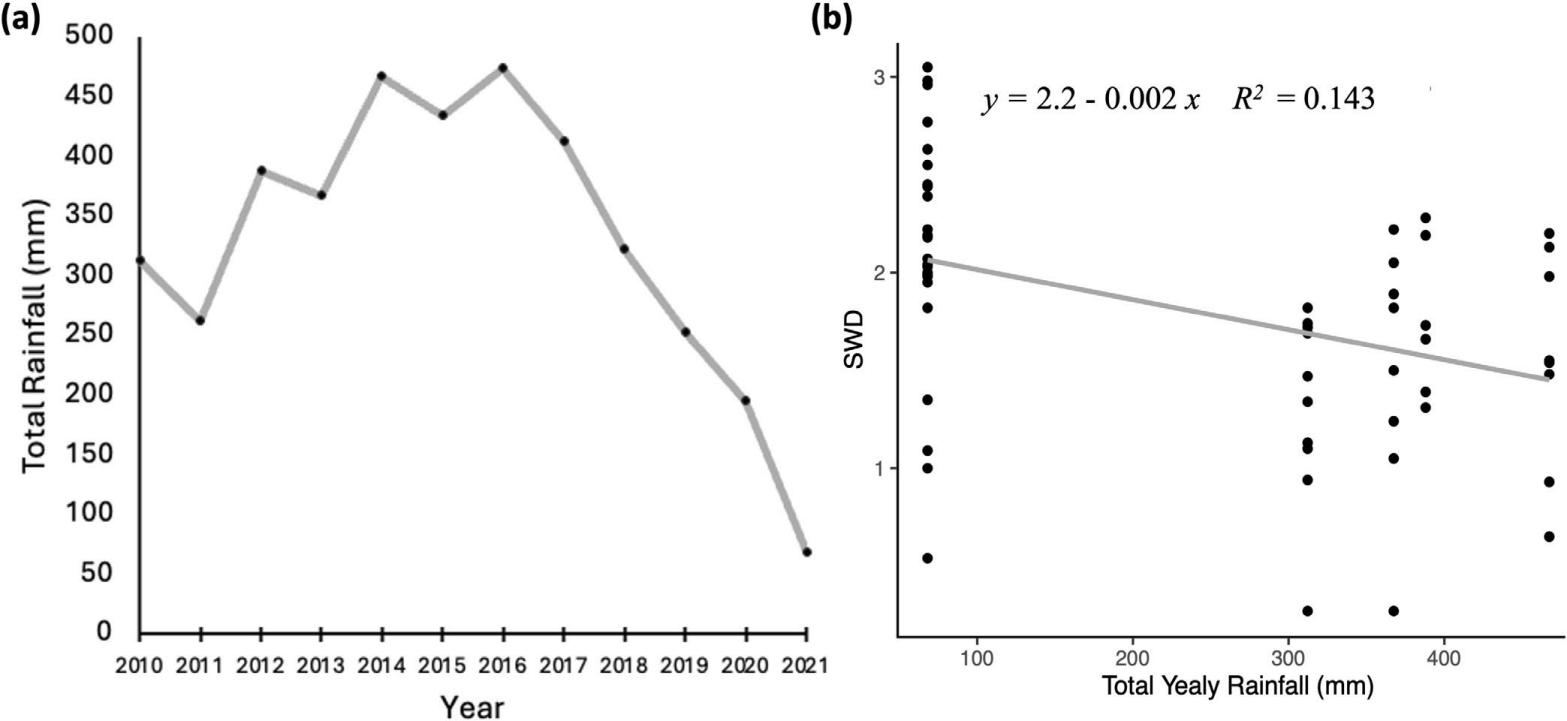
Plots depicting hydroclimate as total rainfall across sampling years (a), and its regression to Shannon‐Weiner diversity (b).

Variation in total abundance and SWD were both influenced by the number of slumps upstream; however, abundance was most influenced by slump variables and SWD was most influenced by differences between years. For abundance, log_10_ TSS (*R* ^2^ = 0.43, *p* = < 0.001), log_10_ number of upstream slumps (*R* ^2^ = 0.17, *p* = 0.002), and active area (*R* ^2^ = 0.17, *p* = 0.002) all explained a significant amount of variation in the dataset (Figure S2), whereas year and distance to the nearest active slump did not (*p* > 0.305). AIC_C_ revealed that the “best” model for explaining trends in abundance included TSS and the number of upstream slumps, where high TSS values and a greater number of upstream slumps both resulted in a decrease in total abundance (adj. *R* ^2^ = 0.43, *p* < 0.001) (Tables S8 and S10). Overall, log_10_ TSS (Cum. *W*_*i*_ = 1) was 1.78 times more important for explaining variation in abundance than the number of slumps upstream (Cum. *W*_*i*_ = 0.56), and 3.22 times more important than active area (Cum. *W*_*i*_ = 0.31). Further, ANCOVA revealed that the slope of the relationship between abundance and TSS, number of upstream slumps, and active area did not differ significantly among sampling periods (interaction *p* > 0.05 for all three models; Figure 6). For SWD, both year (adj. *R* ^2^ = 0.18, *p* < 0.001) and log_10_ number of upstream slumps (adj. *R* ^2^ = 0.11, *p* = 0.007) explained a significant amount of variation (Figure S3), whereas distance to the nearest slump, active area, and log_10_ TSS did not (*p* > 0.324). AICc ranking revealed the “best” model for SWD used both year and number of slumps upstream as predictor variables (adj. *R* ^2^ = 0.26, *p* < 0.001) (Tables S11 and S12), where SWD increased across sampling years and number of upstream slumps (Figure S3). Year (Cum. *W*_*i*_ = 0.99) was 1.06 times more important in explaining variation in SWD than number of slumps upstream (Cum. *W*_*i*_ = 0.93). ANCOVA revealed that the slope of the relationship between SWD and the number of upstream slumps did not differ significantly among sampling periods (interaction *p* = 0.392). Further, hydroclimate had affected SWD, where 2010–14 mean annual rainfall was on average 5.63 times greater than 2021 (Figure 7a), and there was a significant negative relationship between SWD and total yearly rainfall (adj. *R* ^2^ = 0.217; *p* = 0.004; Figure 7b). Overall, models using thaw slump variables as predictors explained more variation (*R* ^2^ range: 0.11–0.43) in total abundance than for SWD (*R* ^2^ range: 0.001–0.13).

## 4 Discussion

Re‐sampling of permafrost thaw slump sites after 10 years indicated that thaw slumps are causing a disturbance‐driven regime change in Arctic streams, as high TSS and nutrient concentrations persisted in 2021. Further, the strongest response of stream invertebrate assemblages to thaw slumps was a pattern of decreasing invertebrate abundance with increasing TSS, where TSS acted as a nonspecific stressor by limiting the abundance of different invertebrate groups, except for disturbance tolerant chironomids. When tested across sites within a slump class, the lack of significant differences in TSS and nutrient concentrations between sampling periods suggested that watersheds impacted by persistent thaw slump activity do not have the capacity to return to an unimpacted state, resulting in chronically elevated “baseline” TSS and nutrient concentrations in RTS streams. Such drastic regime changes have affected stream biotic communities, with invertebrate abundances remaining consistently low in impacted watersheds. However, community composition has changed between sampling periods, as chironomid abundance and chironomid richness were significantly higher on average in 2021 compared to 2010–14. The differences in chironomids between sampling campaigns drove increases in taxonomic diversity (SWD) and evenness evident in 2021. Overall, total suspended solids (as a measure of sediment inputs from thaw slumps) and the number of thaw slumps upstream of sampling sites were important drivers for abundance, and to a lesser extent, taxonomic diversity as thaw slump impacts cascaded downstream.

### 4.1 Thaw Slump Effects on the Abiotic Environment

Thawing permafrost is resulting in a changing disturbance regime in northern watersheds, as thaw slumps have mobilized previously frozen slope materials to nearby stream networks (Kokelj et al. 2015) causing TSS and TP concentrations to remain high over a decade after the initial sampling of the sites assessed in this study. Previous examinations of fluvial regime shifts caused by landscape disturbance have not focused on climate change‐driven changes, such as permafrost degradation in the form of chronic RTS. Rather, past research has largely focused on anthropogenic landscape disturbances such as urbanization (Whitney et al. 2015), agriculture (Beattie et al. 2020; Sutherland et al. 2012; Collier and Quinn 2003; Harding et al. 1998; O'Connor and Lake 1994), and deforestation (Betts et al. 2022; Restrepo et al. 2015; Iwata et al. 2003). While both RTS and anthropogenic disturbances increase TSS and nutrients in impacted stream ecosystems to an elevated baseline, sediment concentrations downstream from thaw slumps (e.g., mean TSS concentration in RTS impacted streams = 2145 mg/L; maximum TSS = 28,500 mg/L) appear to reach greater TSS concentrations as compared to anthropogenic disturbances. For example, streams in agricultural row crop landscapes have mean TSS concentrations of 200 mg/L and may reach concentrations of up to 9000 mg/L if overland flooding occurs (Dodds and Whiles 2004), while stream sites impacted by deforestation may have TSS increases of approximately 21 mg/L in impacted sites as compared to reference sites due to decreases in bank stability and riparian area (de Mello et al. 2018). The difference in intensity of TSS between thaw slump‐impacted streams and the impact from other stressors is partly due to the fine‐grained nature of ice‐rich tills that comprise permafrost in the Peel Plateau. Furthermore, positive feedback associated with debris tongue development raises stream base level, enhancing slope erosion and often causing development of secondary slumps (Kokelj et al. 2021). As ice‐rich headwalls thaw during warm periods, meltwater saturates fine‐grained sediments, causing downslope flow of materials by surface wash, slides, and fluidized flow (Kokelj et al. 2015). The mechanism of thawing transfers sediments directly to streams, which does not typically happen in non‐permafrost systems, resulting in a more intensive disturbance regime in RTS watersheds as compared to other disturbances. Permafrost thaw is projected to continue with climate warming through the coming century (Hu et al. 2022). In ice‐rich regions, thaw slumps will have a high magnitude and persistent impact on streams. These effects will cascade downstream, causing cumulative impacts on larger river systems (Kokelj et al. 2021; Segal et al. 2016). Thus, it is important to quantify the geomorphic dynamics of slumps (activity level), their longevity, and the nature and quantities of sediment transfer over time to understand better how biological communities will respond to the changing conditions.

Unlike other landscape disturbances that are more static in nature, such as damming (Wang et al. 2022; Tullos et al. 2014), river channelization (Ciszewski and Czajka 2015; Simon 1989), and the construction of nearby bridges and roads (Trombulak and Frissell 2000), thaw slumps are dynamic disturbances, producing constant yet varying severity of impact through cyclical periods of high and low activity. For example, thaw slump activity fluctuates annually (i.e., due to seasonal variation; Zwieback et al. 2018) and diurnally (i.e., due to daily variation in solar radiation; Kokelj et al. 2013), with additional activity pulses due to acute disturbance events (i.e., rainstorms; Kokelj et al. 2015, and wildfire; Nesterova et al. 2024). Here, we did not detect any large‐scale TSS change in stream sites relative to 2010–2014 despite a general intensification of slumping in the region, highlighting that slumping is likely so pronounced that chronically high TSS concentrations are approaching the sediment carrying capacity of streams. However, greater variation between some impacted sites during the 2021 sampling campaign remained evident. This was likely because our study design reflected slump‐to‐slump variation in activity and complexity in site‐scale responses. For instance, TSS concentrations had the largest increases at sites in low order streams (i.e., 1–3) located within 2 km of a highly active thaw slump (slump activity > 60%; i.e., STN 38B, 40A, and 40B), as well as in sites located further downstream in the watershed (i.e., STN 21, 24, and 56). Such large differences in TSS likely occurred in these sites over time because small headwater streams that had not reached sediment carrying capacity in 2010–2014 now have, and higher order streams subjected to the chronic exposure of TSS accumulation from multiple upstream thaw slumps are now at or nearing their carrying capacity. Thaw slump disturbances within watersheds create a pattern of disturbance on the landscape. Large spatial scale modeling is needed to predict future impacts on stream ecosystem processes (e.g., biotic productivity), which requires a more detailed understanding of how slump activity accumulates within and is transferred through the watershed.

While sediment input from thaw slumps presents the most dramatic press disturbance to water quality in the Peel Plateau, concentrations of water chemistry variables are not solely dependent on the effects of thaw slump disturbances between sampling campaigns. Discharge and dilution effects can play an important influence on variation in water quality concentrations. For example, significant changes in major ion concentrations (e.g., Mg and Ca) among years were not associated with slump impact. In this case, interannual differences in rainfall may be important as the 2021 sampling season was an abnormally dry year compared to the 2010–2014 period (Kokelj et al. 2022). Edwards (1973) and Reid et al. (1981) show that Na and Mg concentrations are inversely related to rainfall, where increased discharge dilutes major ion concentrations in streams. Further, Sutcliffe and Carrick (1983) show that Mg, Ca, and Na are related to discharge, where high discharge seasons relate to increased major ion flux.

### 4.2 Invertebrate Metrics and Community Composition Across Sampling Periods

Thaw slumps continue to be stressors on invertebrate communities in streams, with their effects being manifested mainly through changes in abundance that are strongly related to TSS concentration and slump classification (i.e., distance to the slump and active area of the slump; Chin et al. 2016; Levenstein et al. 2021). We found lower total, EPT, and Chironomidae abundances at highly impacted sites in 2021, a response which is consistent with the impacts of other chronic disturbances that cause high rates of sedimentation including agriculture (Davis et al. 2022), deforestation (Betts et al. 2022), and managed stream flow (Xu and Li 2019). The strong link between BMI abundance and slump impact may indicate that some recolonization of disturbance‐tolerant invertebrates is possible if slumps become inactive. However, as slumps reactivate, abundance will predictably decrease. Changes in abundance and TSS concentration driven by thaw slumps contrast with other chronic disturbances that show landscape recovery and a reduction in sedimentation effects over shorter time frames (e.g., deforestation and regrowth of riparian forests; Iwata et al. 2003). Thaw slumps may demonstrate recovery trends over longer time frames (i.e., > 50 years; Lantuit and Pollard 2008) once erosive activity ceases, but this has yet to be observed in the Peel Plateau region where there has been a progressive increase in slump area at the watershed scale over the past 30 years (Segal et al. 2016). Further, current research (van der Sluijs et al. 2023) indicates that slumps often become permanent landscape features with a varying severity and potential to revegetate after periods of inactivity (Lantz et al. 2009), and debris tongues enabling persistence of the stress.

Climatic variation (e.g., changes in rainfall) may be associated with compositional changes in invertebrate communities within the limited natural variation observed in high disturbance environments. Taxonomic diversity was higher across all slump classes in 2021 than the 2010–2014 sampling campaigns, a trend that was driven by higher chironomid taxonomic richness in 2021 as compared to 2010–2014. This pattern also occurred in community composition, where compositional differences between 2010–2014 and 2021 were driven by higher average relative abundances of chironomid subfamilies (i.e., Orthocladiinae) in 2021. Diversity and community composition changes may be associated with year‐to‐year variation in environmental factors unrelated or indirectly related to slump activity, as the magnitude of increase in Chironomidae was even across all slump classes (Jourdan et al. 2018). Chironomids are rapid colonizers with short generation times and are typically more tolerant of changes in water chemistry than EPT (Mackey 1977; Armitage et al. 2012), which may enable them to persist through lower oxygen levels as a result of streambed infilling. Further, burrowing dipterans such as chironomids can persist in a wide range of stream water velocities (Oliver 1971; Rader and Belish 1999) and survive in habitats dominated by fine particulate matter (Oliver 1971). Therefore, one possible explanation for the increase in chironomid abundance and taxa richness between sampling campaigns may be related to the lower water levels in 2021 as compared to 2010–2014. We hypothesize that lower than normal rainfall during 2021 produced lower stream discharge and velocity, thereby reducing scouring of the substrate by suspended and saltating sediment particles and allowing chironomids to rapidly repopulate low flow streams at a faster rate than other taxa (Szczerkowska‐Majchrzak et al. 2010). Additionally, Lento et al. (2022) and Goedkoop et al. (2022) have hypothesized that warming temperatures in the Circumpolar Arctic will lead to the range expansion of southern BMI species, thereby increasing BMI diversity in Arctic streams over time. As chironomids are rapid colonizers, significant increases in chironomid abundance and richness may be the first sign of this change. Changes in rainfall and/or upward trending Arctic temperatures may have contributed to a relative increase in chironomid abundance and richness as compared to EPT (whose richness and abundance did not change between campaigns), driving significant shifts in community composition and diversity metrics over time. Shifts in community composition toward taxa with more similar life strategies can be indicative of ecosystem vulnerability to disturbance as a result of decreased functional diversity within the stream environment (Burley et al. 2016). Therefore, increases in chironomid abundance and richness between sampling campaigns may indicate reduced resiliency in Peel Plateau streams if EPT diversity and abundance do not similarly increase in the future.

### 4.3 Key Thaw Slump Variables Most Influential to Benthic Biodiversity

Considering both sampling campaigns, the number of upstream slumps is an important predictor associated with increased variation in invertebrate metrics (i.e., abundance and to a lesser extent, taxonomic diversity). Previously, Chin et al. (2016) had found distance from the nearest active slump to be an important variable for both water chemistry and invertebrate metrics including abundance (Chin et al. 2016). However, continued mapping of the watershed has allowed access to landscape variables that were not previously available, such as the number of upstream slumps, which is an important driver of BMI abundance and diversity. The cumulative number of slumps upstream was likely an important variable because slump density upstream relates to the location of the site within the watershed. Over time, higher order streams (i.e., > 3rd order) have a greater potential to be impacted by the cumulative effects of TSS from all upstream slumps than headwater streams (Kokelj et al. 2021), even if the stream reach is farther from the nearest slump within the catchment (Kokelj et al. 2013). In addition, thaw slumps provide barriers to the dispersal of aquatic organisms (e.g., via drift) by creating large stretches of streambed that are highly embedded with sediments, creating increased habitat fragmentation (Fryirs 2013; Favaro and Lamoureux 2015). If invertebrate abundance and communities are affected by thaw slumps at sites lower in the watershed, recolonization by upstream regions through drift may be less likely due to this habitat fragmentation. Increased fragmentation may also result in reduced resiliency of benthic invertebrate communities to future disturbances, as taxa occupying distinct trophic niches become less able to recolonize downstream environments or may not be present to recolonize if all instream habitat becomes uniform (Burley et al. 2016). Consequently, the cumulative impacts of slumps located in upstream reaches will likely drive abundance and diversity trends in the lower watershed over centennial time scales or greater (Kokelj et al. 2021).

## 5 Conclusions

By comparing results from 2010–2014 to 2021, we documented stream water quality and BMI community trends after 10 years of exposure to thaw slump runoff. The slump‐driven impacts on the physical stream environment and benthic invertebrate communities remained consistent between sampling periods, as indicated by the lack of significant change in environmental variables denoting slump impact (e.g., TSS and nutrient concentrations), with the constancy of the impact indicating that climate‐driven permafrost thaw is resulting in a changing disturbance regime in Arctic streams. Slumps are now the primary driver of sediment mobilization in permafrost preserved ice‐cored moraine. They affect both headwaters and large rivers as this regime shift has caused slope sediment supply to vastly outpace stream transport capacity, with effects that amplify in downstream reaches. Further, invertebrate metrics across both sampling campaigns were affected by upstream slumps. For BMI abundance, the environmental drivers of community change over time were associated with the impacts of suspended sediment load, a nonspecific stressor that limits all invertebrate taxa. Because TSS concentration did not significantly increase between campaigns, there were no observable changes in total abundance apart from a relative increase in the number of Chironomids across all sites. For SWD, other environmental factors in addition to the number of upstream slumps may be important for determining trends, particularly rainfall. Slumps and their impacts will continue to increase in intensity and size if climate continues to warm. Therefore, long‐term monitoring will be required to assess streams that remain unaffected by thaw slump processes as they could serve as refuge areas for metapopulations that can provide recolonization potential for ecosystems damaged by thaw slumps. As the effects of climate‐driven permafrost thaw continue to increase, periodic biomonitoring as advocated by the Arctic Councils' Circumpolar Biodiversity Monitoring Program of freshwaters will be vital for informing conservation policies in the future (Culp et al. 2012; Goedkoop et al. 2022).

## Author Contributions

**Maria Dolan:** data curation, formal analysis, investigation, methodology, visualization, writing – original draft, writing – review and editing. **Jordan Musetta‐Lambert:** conceptualization, funding acquisition, project administration, resources, supervision, writing – review and editing. **Krista S. Chin:** data curation, methodology, writing – review and editing. **Steven V. Kokelj:** conceptualization, data curation, methodology, writing – review and editing. **Suzanne E. Tank:** methodology, writing – review and editing. **Jennifer Lento:** conceptualization, formal analysis, methodology, writing – review and editing. **Michael Power:** funding acquisition, resources, writing – review and editing. **Joseph M. Culp:** conceptualization, funding acquisition, project administration, resources, supervision, writing – review and editing.

## Funding

This work was supported by Northwest Territories Cumulative Impacts Monitoring Program (GNWT CIMP), CIMP 211 and Polar Continental Shelf Program (PCSP) (216‐22, 604‐22, and 626‐21).

## Conflicts of Interest

The authors declare no conflicts of interest.

## Supporting information


**Appendix S1:** gcb70701‐sup‐0001‐AppendixS1.pdf.

## Data Availability

All data used in this study are available in the Open Science Framework repository. The processed data used in the analysis of this study are available at https://doi.org/10.17605/OSF.IO/PD987 and https://doi.org/10.17605/OSF.IO/DB6CS. Code is available at https://doi.org/10.17605/OSF.IO/DB6CS. Raw data is available at https://doi.org/10.17605/OSF.IO/8WAME (for 2010–2012 data), https://doi.org/10.17605/OSF.IO/SPJ96 (for 2013–2014 data), and https://doi.org/10.17605/OSF.IO/ASCRD (for 2021 data).

## References

[gcb70701-bib-0001] Armitage, P. D. , D. Moss , J. F. Wright , and M. T. Furse . 1983. “The Performance of a New Biological Water Quality Score System Based on Macroinvertebrates Over a Wide Range of Unpolluted Running‐Water Sites.” Water Research 17, no. 3: 333–347.

[gcb70701-bib-0002] Armitage, P. D. , L. C. Pinder , and P. S. Cranston , eds. 2012. The Chironomidae: Biology and Ecology of Non‐Biting Midges. Springer Science & Business Media.

[gcb70701-bib-0003] Arscott, D. B. , J. K. Jackson , and E. B. Kratzer . 2006. “Role of Rarity and Taxonomic Resolution in a Regional and Spatial Analysis of Stream Macroinvertebrates.” Journal of the North American Benthological Society 25, no. 4: 977–997.

[gcb70701-bib-0004] Balser, A. W. , J. B. Jones , and R. Gens . 2014. “Timing of Retrogressive Thaw Slump Initiation in the Noatak Basin, Northwest Alaska, USA.” Journal of Geophysical Research ‐ Earth Surface 119, no. 5: 1106–1120.

[gcb70701-bib-0005] Beattie, R. E. , A. Bandla , S. Swarup , and K. R. Hristova . 2020. “Freshwater Sediment Microbial Communities Are Not Resilient to Disturbance From Agricultural Land Runoff.” Frontiers in Microbiology 11: 539921. 33178143 10.3389/fmicb.2020.539921 PMC7593329

[gcb70701-bib-0006] Betts, J. T. , G. R. Urquhart , J. Roman‐Heracleo , and J. C. Flores Mc. rea . 2022. “Effects of Deforestation From Cattle Ranching Over Time on Protected Rainforest Streams in the Rama‐Kriol Territory, Nicaragua.” Hydrobiologia 849, no. 20: 4547–4568.

[gcb70701-bib-0007] Bilotta, G. S. , and R. E. Brazier . 2008. “Understanding the Influence of Suspended Solids on Water Quality and Aquatic Biota.” Water Research 42, no. 12: 2849–2861. 18462772 10.1016/j.watres.2008.03.018

[gcb70701-bib-0008] Biskaborn, B. K. , U. Herzschuh , D. Y. Bolshiyanov , G. Schwamborn , and B. Diekmann . 2013. “Thermokarst Processes and Depositional Events in a Tundra Lake, Northeastern Siberia.” Permafrost and Periglacial Processes 24, no. 3: 160–174.

[gcb70701-bib-0009] Burley, H. M. , K. Mokany , S. Ferrier , S. W. Laffan , K. J. Williams , and T. D. Harwood . 2016. “Macroecological Scale Effects of Biodiversity on Ecosystem Functions Under Environmental Change.” Ecology and Evolution 6, no. 8: 2579–2593. 27066246 10.1002/ece3.2036 PMC4798165

[gcb70701-bib-0010] Chin, K. S. , J. Lento , J. M. Culp , D. Lacelle , and S. V. Kokelj . 2016. “Permafrost Thaw and Intense Thermokarst Activity Decreases Abundance of Stream Benthic Macroinvertebrates.” Global Change Biology 22, no. 8: 2715–2728. 26766394 10.1111/gcb.13225

[gcb70701-bib-0011] Ciszewski, D. , and A. Czajka . 2015. “Human‐Induced Sedimentation Patterns of a Channelized Lowland River.” Earth Surface Processes and Landforms 40, no. 6: 783–795.

[gcb70701-bib-0012] Cohen, R. S. , D. K. Gray , J. M. Vucic , A. D. Murdoch , and S. Sharma . 2021. “Environmental Variables Associated With Littoral Macroinvertebrate Community Composition in Arctic Lakes.” Canadian Journal of Fisheries and Aquatic Sciences 78, no. 2: 110–123.

[gcb70701-bib-0013] Collier, K. J. , and J. M. Quinn . 2003. “Land‐Use Influences Macroinvertebrate Community Response Following a Pulse Disturbance.” Freshwater Biology 48, no. 8: 1462–1481.

[gcb70701-bib-0014] Culp, J. M. , J. Lento , W. Goedkoop , et al. 2012. “Developing a Circumpolar Monitoring Framework for Arctic Freshwater Biodiversity.” Biodiversity 13: 215–227.

[gcb70701-bib-0015] Culp, J. M. , F. J. Wrona , and R. W. Davies . 1986. “Response of Stream Benthos and Drift to Fine Sediment Deposition Versus Transport.” Canadian Journal of Zoology 64: 1345–1351.

[gcb70701-bib-0016] Davis, N. G. , R. Hodson , and C. D. Matthaei . 2022. “Long‐Term Variability in Deposited Fine Sediment and Macroinvertebrate Communities Across Different Land‐Use Intensities in a Regional Set of New Zealand Rivers.” New Zealand Journal of Marine and Freshwater Research 56, no. 2: 191–212.

[gcb70701-bib-0017] de Mello, K. , R. A. Valente , T. O. Randhir , and C. A. Vettorazzi . 2018. “Impacts of Tropical Forest Cover on Water Quality in Agricultural Watersheds in Southeastern Brazil.” Ecological Indicators 93: 1293–1301.

[gcb70701-bib-0018] Dodds, W. K. , and M. R. Whiles . 2004. “Quality and Quantity of Suspended Particles in Rivers: Continent‐Scale Patterns in the United States.” Environmental Management 33: 355–367. 15031760 10.1007/s00267-003-0089-z

[gcb70701-bib-0019] Dolan, M. 2026. “Retrogressive Thaw Slumps Produce a Changing Disturbance Regime for Arctic Stream Invertebrates.” 10.17605/OSF.IO/PD987. PMC12970577 41801227

[gcb70701-bib-0020] Edwards, A. M. C. 1973. “The Variation of Dissolved Constituents With Discharge in Some Norfolk Rivers.” Journal of Hydrology 18, no. 3–4: 219–242.

[gcb70701-bib-0021] Environment and Climate Change Canada . 2020. National Lab for Environmental Testing Method Descriptions. Environmental Science and Technology Laboratories Version 1. Environment and Climate Change Canada.

[gcb70701-bib-0022] Environment Canada . 2012. Canadian Aquatic Biomonitoring Network Field Manual – Wadeable Streams. Environment Canada.

[gcb70701-bib-0023] Favaro, E. A. , and S. F. Lamoureux . 2015. “Downstream Patterns of Suspended Sediment Transport in a High Arctic River Influenced by Permafrost Disturbance and Recent Climate Change.” Geomorphology 246: 359–369.

[gcb70701-bib-0024] Fox, J. , and S. Weisberg . 2019. An R Companion to Applied Regression_. 3rd ed. Sage, Thousand Oaks CA.

[gcb70701-bib-0025] Fryirs, K. 2013. “(Dis) Connectivity in Catchment Sediment Cascades: A Fresh Look at the Sediment Delivery Problem.” Earth Surface Processes and Landforms 38, no. 1: 30–46.

[gcb70701-bib-0026] Goedkoop, W. , J. M. Culp , T. Christensen , et al. 2022. “Improving the Framework for Assessment of Ecological Change in the Arctic: A Circumpolar Synthesis of Freshwater Biodiversity.” Freshwater Biology 67: 210–223.

[gcb70701-bib-0027] Harding, J. S. , E. F. Benfield , P. V. Bolstad , G. S. Helfman , and E. B. D. Jones Iii . 1998. “Stream Biodiversity: The Ghost of Land Use Past.” Proceedings of the National Academy of Sciences 95, no. 25: 14843–14847. 10.1073/pnas.95.25.14843 PMC24537 9843977

[gcb70701-bib-0028] Hu, G. , L. Zhao , T. Wu , et al. 2022. “Continued Warming of the Permafrost Regions Over the Northern Hemisphere Under Future Climate Change.” Earth's Future 10, no. 9: e2022EF002835.

[gcb70701-bib-0029] Iwata, T. , S. Nakano , and M. Inoue . 2003. “Impacts of Past Riparian Deforestation on Stream Communities in a Tropical Rain Forest in Borneo.” Ecological Applications 13, no. 2: 461–473.

[gcb70701-bib-0030] Jones, J. I. , A. L. Collins , P. S. Naden , and D. A. Sear . 2012. “The Relationship Between Fine Sediment and Macrophytes in Rivers.” River Research and Applications 28, no. 7: 1006–1018.

[gcb70701-bib-0031] Jones, J. I. , J. F. Murphy , A. L. Collins , D. A. Sear , P. S. Naden , and P. D. Armitage . 2012. “The Impact of Fine Sediment on Macro‐Invertebrates.” River Research and Applications 28, no. 8: 1055–1071.

[gcb70701-bib-0032] Jourdan, J. , R. B. O'Hara , R. Bottarin , et al. 2018. “Effects of Changing Climate on European Stream Invertebrate Communities: A Long‐Term Data Analysis.” Science of the Total Environment 621: 588–599. 29195206 10.1016/j.scitotenv.2017.11.242

[gcb70701-bib-0033] Kendrick, M. R. , A. D. Huryn , W. B. Bowden , et al. 2018. “Linking Permafrost Thaw to Shifting Biogeochemistry and Food Web Resources in an Arctic River.” Global Change Biology 24, no. 12: 5738–5750. 30218544 10.1111/gcb.14448

[gcb70701-bib-0034] Kokelj, S. A. , C. R. Beel , R. F. Connon , C. E. D. Graydon , S. V. Kokelj , and C. R. Burn . 2022. Peel Plateau Climate Data, Northwest Territories. Northwest Territories Geological Survey. 10.46887/2022-005.

[gcb70701-bib-0036] Kokelj, S. V. , J. Kokoszka , J. van Der Sluijs , et al. 2021. “Thaw‐Driven Mass Wasting Couples Slopes With Downstream Systems, and Effects Propagate Through Arctic Drainage Networks.” Cryosphere 15, no. 7: 3059–3081.

[gcb70701-bib-0037] Kokelj, S. V. , D. Lacelle , T. C. Lantz , et al. 2013. “Thawing of Massive Ground Ice in Mega Slumps Drives Increases in Stream Sediment and Solute Flux Across a Range of Watershed Scales.” Journal of Geophysical Research. Earth Surface 118, no. 2: 681–692.

[gcb70701-bib-0039] Kokelj, S. V. , T. C. Lantz , J. Tunnicliffe , R. Segal , and D. Lacelle . 2017. “Climate‐Driven Thaw of Permafrost Preserved Glacial Landscapes, Northwestern Canada.” Geology 45, no. 4: 371–374.

[gcb70701-bib-0040] Kokelj, S. V. , J. Tunnicliffe , D. Lacelle , T. C. Lantz , K. S. Chin , and R. Fraser . 2015. “Increased Precipitation Drives Mega Slump Development and Destabilization of Ice‐Rich Permafrost Terrain, Northwestern Canada.” Global and Planetary Change 129: 56–68.

[gcb70701-bib-0041] Lacelle, D. , J. Bjornson , and B. Lauriol . 2010. “Climatic and Geomorphic Factors Affecting Contemporary (1950–2004) Activity of Retrogressive Thaw Slumps on the Aklavik Plateau, Richardson Mountains, NWT, Canada.” Permafrost and Periglacial Processes 21, no. 1: 1–15.

[gcb70701-bib-0042] Lacelle, D. , A. Brooker , R. H. Fraser , and S. V. Kokelj . 2015. “Distribution and Growth of Thaw Slumps in the Richardson Mountains–Peel Plateau Region, Northwestern Canada.” Geomorphology 235: 40–51.

[gcb70701-bib-0043] Lantuit, H. , and W. H. Pollard . 2008. “Fifty Years of Coastal Erosion and Retrogressive Thaw Slump Activity on Herschel Island, Southern Beaufort Sea, Yukon Territory, Canada.” Geomorphology 95, no. 1–2: 84–102.

[gcb70701-bib-0044] Lantz, T. C. , S. V. Kokelj , S. E. Gergel , and G. H. Henry . 2009. “Relative Impacts of Disturbance and Temperature: Persistent Changes in Microenvironment and Vegetation in Retrogressive Thaw Slumps.” Global Change Biology 15, no. 7: 1664–1675.

[gcb70701-bib-0045] Larsen, S. , and S. J. Ormerod . 2010. “Low‐Level Effects of Inert Sediments on Temperate Stream Invertebrates.” Freshwater Biology 55, no. 2: 476–486.

[gcb70701-bib-0046] Lemly, A. D. 1982. “Modification of Benthic Insect Communities in Polluted Streams: Combined Effects of Sedimentation and Nutrient Enrichment.” Hydrobiologia 87, no. 3: 229–245.

[gcb70701-bib-0047] Lenat, D. R. , D. L. Penrose , and K. W. Eagleson . 1981. “Variable Effects of Sediment Addition on Stream Benthos.” Hydrobiologia 79, no. 2: 187–194.

[gcb70701-bib-0048] Lento, J. , J. M. Culp , B. Levenstein , et al. 2022. “Temperature and Spatial Connectivity Drive Patterns in Freshwater Macroinvertebrate Diversity Across the Arctic.” Freshwater Biology 67, no. 1: 159–175.

[gcb70701-bib-0049] Levenstein, B. , J. M. Culp , and J. Lento . 2018. “Sediment Inputs From Retrogressive Thaw Slumps Drive Algal Biomass Accumulation but Not Decomposition in Arctic Streams, NWT.” Freshwater Biology 63, no. 10: 1300–1315.

[gcb70701-bib-0050] Levenstein, B. , J. Lento , and J. Culp . 2021. “Effects of Prolonged Sedimentation From Permafrost Degradation on Macroinvertebrate Drift in Arctic Streams.” Limnology and Oceanography 66: S157‐S168.

[gcb70701-bib-0089] Lewkowicz, A. G. , and R. G. Way . 2018. “Extremes of Summer Climate Trigger Thousands of Thermokarst Landslides in a High Arctic Environment.” Nature Communications 10, no. 1: 1329. 10.1038/s41467-019-09314-7 PMC6445831 30940802

[gcb70701-bib-0051] Luo, J. , F. Niu , Z. Lin , M. Liu , and G. Yin . 2019. “Recent Acceleration of Thaw Slumping in Permafrost Terrain of Qinghai‐Tibet Plateau: An Example From the Beiluhe Region.” Geomorphology 341: 79–85.

[gcb70701-bib-0052] Mackey, A. P. 1977. “Growth and Development of Larval Chironomidae.” Oikos 28: 270–275.

[gcb70701-bib-0054] Molineri, C. , E. G. Tejerina , S. E. Torrejón , E. J. Pero , and G. E. Hankel . 2020. “Indicative Value of Different Taxonomic Levels of Chironomidae for Assessing the Water Quality.” Ecological Indicators 108: 105703.

[gcb70701-bib-0055] Moquin, P. A. , P. S. Mesquita , F. J. Wrona , and T. D. Prowse . 2014. “Responses of Benthic Invertebrate Communities to Shoreline Retrogressive Thaw Slumps in Arctic Upland Lakes.” Freshwater Science 33, no. 4: 1108–1118.

[gcb70701-bib-0057] Mustonen, K. R. , H. Mykrä , P. Louhi , et al. 2016. “Sediments and Flow Have Mainly Independent Effects on Multitrophic Stream Communities and Ecosystem Functions.” Ecological Applications 26, no. 7: 2116–2129. 27755727 10.1890/15-1841.1

[gcb70701-bib-0058] Nesterova, N. , M. Leibman , A. Kizyakov , et al. 2024. “Retrogressive Thaw Slump Characteristics and Terminology.” Cryosphere 18, no. 10: 4787–4810.

[gcb70701-bib-0059] Nilsson, C. , L. E. Polvi , and L. Lind . 2015. “Extreme Events in Streams and Rivers in Arctic and Subarctic Regions in an Uncertain Future.” Freshwater Biology 60, no. 12: 2535–2546.

[gcb70701-bib-0060] O'Connor, N. A. , and P. S. Lake . 1994. “Long‐Term and Seasonal Large‐Scale Disturbances of a Small Lowland Stream.” Marine and Freshwater Research 45, no. 2: 243–255.

[gcb70701-bib-0061] O'Donnell, J. A. , M. P. Carey , J. C. Koch , et al. 2020. “Permafrost Hydrology Drives the Assimilation of Old Carbon by Stream Food Webs in the Arctic.” Ecosystems 23: 435–453.

[gcb70701-bib-0062] Oksanen, J. , G. Simpson , F. Blanchet , et al. 2024. “vegan: Community Ecology Package.” R Package Version 2.6–8. https://CRAN.R‐project.org/package=vegan.

[gcb70701-bib-0063] Oliver, D. R. 1971. “Life History of the Chironomidae.” Annual Review of Entomology 16, no. 1: 211–230.

[gcb70701-bib-0064] O'Neill, H. B. , C. R. Burn , S. V. Kokelj , and T. C. Lantz . 2015. “‘Warm’ Tundra: Atmospheric and Near‐Surface Ground Temperature Inversions Across an Alpine Treeline in Continuous Permafrost, Western Arctic, Canada.” Permafrost and Periglacial Processes 26, no. 2: 103–118.

[gcb70701-bib-0065] R Core Team . 2024. R: A Language and Environment for Statistical Computing. R Foundation for Statistical Computing.

[gcb70701-bib-0066] Rader, R. B. , and T. A. Belish . 1999. “Influence of Mild to Severe Flow Alterations on Invertebrates in Three Mountain Streams.” Regulated Rivers: Research & Management: An International Journal Devoted to River Research and Management 15, no. 4: 353–363.

[gcb70701-bib-0067] Raunio, J. , R. Paavola , and T. Muotka . 2007. “Effects of Emergence Phenology, Taxa Tolerances and Taxonomic Resolution on the Use of the Chironomid Pupal Exuvial Technique in River Biomonitoring.” Freshwater Biology 52, no. 1: 165–176.

[gcb70701-bib-0068] Reid, J. M. , D. A. MacLeod , and M. S. Cresser . 1981. “Factors Affecting the Chemistry of Precipitation and River Water in an Upland Catchment.” Journal of Hydrology 50: 129–145.

[gcb70701-bib-0069] Restrepo, J. D. , A. J. Kettner , and J. P. Syvitski . 2015. “Recent Deforestation Causes Rapid Increase in River Sediment Load in the Colombian Andes.” Anthropocene 10: 13–28.

[gcb70701-bib-0070] Segal, R. A. , T. C. Lantz , and S. V. Kokelj . 2016. “Acceleration of Thaw Slump Activity in Glaciated Landscapes of the Western Canadian Arctic.” Environmental Research Letters 11, no. 3: e034025.

[gcb70701-bib-0071] Séjourné, A. , F. Costard , A. Fedorov , et al. 2015. “Evolution of the Banks of Thermokarst Lakes in Central Yakutia (Central Siberia) due to Retrogressive Thaw Slump Activity Controlled by Insolation.” Geomorphology 241: 31–40.

[gcb70701-bib-0072] Shakil, S. , S. E. Tank , S. V. Kokelj , J. E. Vonk , and S. Zolkos . 2020. “Particulate Dominance of Organic Carbon Mobilization From Thaw Slumps on the Peel Plateau, NT: Quantification and Implications for Stream Systems and Permafrost Carbon Release.” Environmental Research Letters 15, no. 11: 114019.

[gcb70701-bib-0073] Simon, A. 1989. “The Discharge of Sediment in Channelized Alluvial Streams 1.” JAWRA Journal of the American Water Resources Association 25, no. 6: 1177–1188.

[gcb70701-bib-0074] Strong, W. L. 2016. “Biased Richness and Evenness Relationships Within Shannon–Wiener Index Values.” Ecological Indicators 67: 703–713.

[gcb70701-bib-0090] Sutcliffe, D. W. , and T. R. Carrick . 1983. “Relationships Between Chloride and Major Cations in Precipitation and Streamwaters in the Windermere Catchment (English Lake District).” Freshwater Biology 13, no. 5: 415–441.

[gcb70701-bib-0075] Sutherland, A. B. , J. M. Culp , and G. A. Benoy . 2012. “Evaluation of Deposited Sediment and Macroinvertebrate Metrics Used to Quantify Biological Response to Excessive Sedimentation in Agricultural Streams.” Environmental Management 50: 50–63. 22525990 10.1007/s00267-012-9854-1

[gcb70701-bib-0076] Symonds, M. R. , and A. Moussalli . 2011. “A Brief Guide to Model Selection, Multimodel Inference and Model Averaging in Behavioural Ecology Using Akaike's Information Criterion.” Behavioral Ecology and Sociobiology 65, no. 1: 13–21.

[gcb70701-bib-0077] Szczerkowska‐Majchrzak, E. , M. Grzybkowska , and M. Dukowska . 2010. “Effect of Flow Fluctuations on Patch Dynamics and Chironomid Distribution in a Medium‐Sized Lowland River.” Journal of Freshwater Ecology 25, no. 3: 437–448.

[gcb70701-bib-0078] Trombulak, S. C. , and C. A. Frissell . 2000. “Review of Ecological Effects of Roads on Terrestrial and Aquatic Communities.” Conservation Biology 14, no. 1: 18–30.

[gcb70701-bib-0079] Tullos, D. D. , D. S. Finn , and C. Walter . 2014. “Geomorphic and Ecological Disturbance and Recovery From Two Small Dams and Their Removal.” PLoS One 9, no. 9: e108091. 25233231 10.1371/journal.pone.0108091 PMC4169487

[gcb70701-bib-0080] van der Sluijs, J. , S. V. Kokelj , and J. F. Tunnicliffe . 2023. “Allometric Scaling of Retrogressive Thaw Slumps.” Cryosphere 17, no. 11: 4511–4533.

[gcb70701-bib-0081] Vonk, J. E. , S. E. Tank , W. B. Bowden , et al. 2015. “Reviews and Syntheses: Effects of Permafrost Thaw on Arctic Aquatic Ecosystems.” Biogeosciences 12, no. 23: 7129–7167.

[gcb70701-bib-0082] Wang, X. , Y. Chen , Q. Yuan , et al. 2022. “Effect of River Damming on Nutrient Transport and Transformation and Its Countermeasures.” Frontiers in Marine Science 9: 1078216.

[gcb70701-bib-0083] Whitney, J. W. , P. A. Glancy , S. E. Buckingham , and A. C. Ehrenberg . 2015. “Effects of Rapid Urbanization on Streamflow, Erosion, and Sedimentation in a Desert Stream in the American Southwest.” Anthropocene 10: 29–42.

[gcb70701-bib-0084] Wickham, H. 2016. ggplot2: Elegant Graphics for Data Analysis. Springer‐Verlag New York.

[gcb70701-bib-0085] Wickham, H. , R. François , L. Henry , K. Müller , and D. Vaughan . 2023. “dplyr: A Grammar of Data Manipulation.” R Package Version 1.1.4. https://CRAN.R‐project.org/package=dplyr.

[gcb70701-bib-0086] Wlodarska‐Kowalczuk, M. , T. H. Pearson , and M. A. Kendall . 2005. “Benthic Response to Chronic Natural Physical Disturbance by Glacial Sedimentation in an Arctic Fjord.” Marine Ecology Progress Series 303: 31–41.

[gcb70701-bib-0087] Xu, C. , and Y. Li . 2019. “Effect of Flow‐Sediment Regime on Benthic Invertebrate Communities: Long‐Term Analysis in a Regulated Floodplain Lake.” Science of the Total Environment 649: 201–211. 30173029 10.1016/j.scitotenv.2018.08.308

[gcb70701-bib-0088] Zwieback, S. , S. V. Kokelj , F. Günther , J. Boike , G. Grosse , and I. Hajnsek . 2018. “Sub‐Seasonal Thaw Slump Mass Wasting Is Not Consistently Energy Limited at the Landscape Scale.” Cryosphere 12, no. 2: 549–564.

