# Supplementary material for: Retrogressive Thaw Slumps Produce a Changing Disturbance Regime for Arctic Stream Invertebrates
Source: Glob Chang Biol. 2026 Mar 9;32(3):e70701. doi: 10.1111/gcb.70701 (PMC12970577; doi:10.1111/gcb.70701)
Supplement: Supplementary file 1 — Appendix S1: gcb70701‐sup‐0001‐AppendixS1.pdf. [file GCB-32-e70701-s001.pdf]

Supplemental Materials
Tables

Table S.1. Site code and the years in which the site was sampled.

Site	2010	2012	2013	2014	2021
STN-02	x				x
STN-21	x				x
STN-24	x				x
STN-26	x				x
STN-28	x				x
STN-30-A	x		x		x
STN-30-B			x		x
STN-35	x				x
STN-36	x				x
STN-38-A				x	x
STN-38-B	x		x	x	x
STN-40-A	x		x	x	x
STN-40-B			x	x	x
STN-48		x			x
STN-50		x			x
STN-53		x			x
STN-56		x			x
STN-58		x			x
STN-66		x			x
STN-67		x			x
STN-71-A			x	x	x
STN-71-B			x	x	x
STN-76-A				x	x
STN-76-B				x	x

Table S.2. Taxonomic resolution of benthic invertebrates collected from CABIN kick net samples. Each of the listed taxa were collected at a minimum of one site and utilised in multivariate benthic assemblage analysis including Non-metric multidimensional scaling (NMDS), Permutational analysis of variance (PERMANOVA), and similarity percentage (SIMPER).

Phylum	Class	Order	Family	Subfamily
Arthropoda	Insecta	Ephemeroptera	Baetidae	
			Ephemerellidae	
			Heptageniidae	
			Leptophlebiidae	
		Plecoptera	Capniidae	
			Chloroperlidae	
			Nemouridae	
			Perlodidae	
		Trichoptera	Brachycentridae	
			Glossosomatidae	
			Limnephilidae	
			Rhyacophilidae	
		Diptera	Ceratopogonidae	
			Chironomidae	Chironominae
				Diamesinae
				Prodiamesinae
				Orthocladiinae
				Tanypodinae
				Podonominae
			Dixidae	
			Dolichopodidae	
			Empididae	
			Limoniidae	
			Muscidae	
			Psychodidae	
			Sciaridae	
			Simuliidae	
			Tipulidae	
		Coleoptera	Carabidae	
			Curculionidae	
			Dytiscidae	
			Elmidae	
			Hydrophilidae	
			Staphylinidae	
	Arachnida	Trombidiformes	Hygrobatidae	
			Lebertiidae	
			Sperchontidae	
		Oribatida	Hydrozetidae	
	Malacostraca	Amphipoda	Crangonyctidae	
	Oligochaeta	Lumbriculida	Lumbriculidae	
		Tubificida	Naididae	

Table S.3. Corresponding analysis of variance (ANOVA) tables for significant water chemistry variables demonstrated in Fig 2. Significance indicated by *.

Log10 TSS Anova

Term	Df	Sum Sq	Mean Sq	F value	Pr(>F)
Year	1	2.01	2.011	1.447	0.23606
Slump Class	3	20.30	6.768	4.869	0.00558*
Year*Slump Class	3	1.10	0.367	0.264	0.85097
Residuals	40	55.60	1.390	-	-

Log10 TP Anova

Term	Df	Sum Sq	Mean Sq	F value	Pr(>F)
Year	1	0.01	0.007	0.007	0.933217
Slump Class	3	24.12	8.040	7.938	0.000286*
Year*Slump Class	3	1.39	0.464	0.458	0.713179
Residuals	40	40.52	1.013	-	-

Log10 TN Anova

Term	Df	Sum Sq	Mean Sq	F value	Pr(>F)
Year	1	1.142	1.1424	3.140	0.0840
Slump Class	3	3.839	1.2796	3.517	0.0236*
Year*Slump Class	3	0.078	0.0260	0.071	0.9749
Residuals	40	14.554	0.3638	-	-

Log10 Mg Anova

Term	Df	Sum Sq	Mean Sq	F value	Pr(>F)
Year	1	1.208	1.2084	7.134	0.0109*
Slump Class	3	0.269	0.0897	0.530	0.6645
Year*Slump Class	3	0.031	0.0105	0.062	0.9796
Residuals	40	6.775	0.1694	-	-

Log10 Ca Anova

Term	Df	Sum Sq	Mean Sq	F value	Pr(>F)
Year	1	0.947	0.9471	7.122	0.0109*
Slump Class	3	0.307	0.1025	0.771	0.5173
Year*Slump Class	3	0.016	0.0054	0.041	0.9888
Residuals	40	5.319	0.1330	-	-

Table S.4. Corresponding analysis of variance (ANOVA) tables for significant invertebrate metrics demonstrated in Fig 3. Significance indicated by *.

Log10 Abundance Anova

Term	Df	Sum Sq	Mean Sq	F value	Pr(>F)
Year	1	0.094	0.0935	0.246	0.622317
Slump Class	3	9.161	3.0538	8.048	0.000259*
Year*Slump Class	3	0.107	0.0355	0.094	0.963095
Residuals	40	15.178	0.3795	-	-

Log10 Chironomid Abundance Anova

Term	Df	Sum Sq	Mean Sq	F value	Pr(>F)
Year	1	3.148	3.148	5.818	0.020541*
Slump Class	3	12.359	4.120	7.614	0.000384*
Year*Slump Class	3	0.361	0.120	0.222	0.880416
Residuals	40	21.641	0.541	-	-

SWD Anova

Term	Df	Sum Sq	Mean Sq	F value	Pr(>F)
Year	1	3.546	3.546	9.423	0.00384*
Slump Class	3	0.096	0.032	0.085	0.96801
Year*Slump Class	3	1.439	0.480	1.274	0.29622
Residuals	40	15.054	0.376	-	-

Evenness Anova

Term	Df	Sum Sq	Mean Sq	F value	Pr(>F)
Year	1	0.1939	0.19386	5.291	0.0267*
Slump Class	3	0.0120	0.00402	0.110	0.9540
Year*Slump Class	3	0.1157	0.03857	1.053	0.3799
Residuals	40	1.4656	0.03664	-	-

Total Richness Anova

Term	Df	Sum Sq	Mean Sq	F value	Pr(>F)
Year	1	444.1	444.1	8.071	0.00704*
Slump Class	3	194.2	64.7	1.177	0.33075
Year*Slump Class	3	125.5	41.8	0.760	0.52318
Residuals	40	2200.9	55.0	-	-

Chironomid Richness Anova

Term	Df	Sum Sq	Mean Sq	F value	Pr(>F)
Year	1	238.5	238.52	25.107	1.14e-05*
Slump Class	3	44.5	14.83	1.561	0.214
Year*Slump Class	3	54.8	18.27	1.923	0.141
Residuals	40	380.0	9.50	-	-

Table S.5. Permutational analysis of variance (PERMANOVA) table for sites described by invertebrate metrics. Sites dispersed distinctly according to year (2010-14 and 2021).

Term	DF	Sum of Sqs	R2	F	Pr(>F)
Year	1	0.43286	0.19277	10.7056	0.001
Slump Class	3	0.13833	0.06160	1.1404	0.328
Year * Slump Class	3	0.05693	0.02535	0.4693	0.894
Residual	40	1.61732	0.72027	-	-
Total	47	2.24545	1.00000	-	-

Table S.6. Permutational analysis of variance (PERMANOVA) table for sites summarized by the relative abundance of common invertebrate families (community composition). Sites dispersed distinctly according to year (2010-14 and 2021).

Term	DF	Sum of Sqs	R2	F	Pr(>F)
Year	1	1.0014	0.10382	5.1592	0.001
Slump Class	3	0.4601	0.04770	0.7902	0.730
Year*Slump Class	3	0.4202	0.04356	0.7216	0.791
Residual	40	7.7640	0.80492	-	-
Total	47	9.6456	1.00000	-	-

Table S.7. Similarity percentage (SIMPER) table for invertebrate summary metrics that drove differences between years in NMDS. average = average contribution of the metric to the average dissimilarity between observations from the two groups. SD = standard deviation of the contribution of the metric. CV= coefficient of variation. 2021 Avg, 2010-2014 Avg = average value for the metric in each of the two sampling periods. Cum Cont = cumulative contribution of this and all previous metrics in list (Based on average but expressed as a proportion of the average dissimilarity). p = permutation-based p-value; probability of getting a larger or equal average contribution for each metric if the grouping factor was randomly permuted.

Metric	Average	SD	CV	2021 Avg	2010-14 Avg	Cum Cont	p
Richness	0.13428	0.08873	1.51350	23.87500	15.25000	0.436	0.001
Chironomidae Richness	0.06040	0.04107	1.47080	8.79200	4.20800	0.632	0.001
Ephemeroptera Richness	0.02388	0.01908	1.25170	2.29200	2.12500	0.709	0.761
Plecoptera Richness	0.01942	0.01522	1.27560	3.08300	2.79200	0.772	0.993
Log_{10} Chironomid Abundance	0.01265	0.00993	1.27340	2.50400	1.93600	0.813	0.084
Log_{10} EPT Abundance	0.01193	0.00939	1.27020	1.90000	2.02800	0.852	0.320
Log_{10} Total Abundance	0.01050	0.00821	1.27920	2.62100	2.49700	0.886	0.993
Trichoptera Richness	0.00999	0.01171	0.85350	0.58300	0.58300	0.919	1.000
SWD	0.00987	0.00737	1.33940	2.11100	1.60700	0.951	0.002
Percent Chironomidae	0.00510	0.00383	1.33180	0.49600	0.20800	0.967	0.010
Percent Plecoptera	0.00303	0.00285	1.06390	0.25800	0.18800	0.977	0.639
Percent Ephemeroptera	0.00266	0.00280	0.95040	0.06600	0.23800	0.986	0.131
Evenness	0.00263	0.00224	1.17300	0.68100	0.60800	0.994	0.251
Percent Trichoptera	0.00179	0.00342	0.52260	0.01400	0.13800	1.000	0.984

Table S.8. Similarity percentage (SIMPER) table for invertebrate taxa that drove differences between years in community composition NMDS. average = average contribution of the taxa to the average dissimilarity between observations from the two groups. SD = standard deviation of the contribution of the taxa. CV= coefficient of variation. 2021 Avg, 2010-2014 Avg = average value for the metric in each of the two sampling periods. Cum Cont = cumulative contribution of this and all previous taxa in list (Based on average but expressed as a proportion of the average dissimilarity). p = permutation-based p-value; probability of getting a larger or equal average contribution for each taxon if the grouping factor was randomly permuted.

Taxa	Average	SD	CV	2021 Avg	2010-14 Avg	Cum Cont	p
Chironomidae	0.10338	0.06850	1.50920	0.31425	0.17755	0.162	0.002
Nemouridae	0.09852	0.10288	0.95760	0.18516	0.15308	0.315	0.714
Orthocladiinae	0.08898	0.06697	1.32860	0.22371	0.08460	0.454	0.001
Heptageniidae	0.06587	0.09924	0.66370	0.01632	0.12784	0.557	0.003
Simuliidae	0.06330	0.11767	0.53790	0.01136	0.12086	0.656	0.021
Diamesinae	0.03781	0.05430	0.69620	0.03492	0.05992	0.715	0.406
Capniidae	0.03679	0.06501	0.56580	0.02159	0.06010	0.773	0.131
Baetidae	0.03256	0.04487	0.72570	0.02647	0.06020	0.824	0.188
Trombidiformes	0.01794	0.03794	0.47290	0.01373	0.03097	0.852	0.670
Chironominae	0.01760	0.03104	0.56680	0.02862	0.01067	0.879	0.084
Tipulidae	0.01500	0.01748	0.85810	0.03296	0.00987	0.902	0.003
Lumbriculidae	0.01461	0.01607	0.90940	0.00610	0.02838	0.925	0.003
Sperchontidae	0.00859	0.01993	0.43100	0.00643	0.01256	0.939	0.796
Ephemerellidae	0.00713	0.01939	0.36800	0.00944	0.00523	0.950	0.776
Brachycentridae	0.00560	0.00962	0.58250	0.00843	0.00399	0.959	0.414
Ceratopogonidae	0.00537	0.01435	0.37450	0.00874	0.00230	0.967	0.133
Podonominae	0.00431	0.00909	0.47420	0.00516	0.00377	0.974	0.068
Oribatida	0.00415	0.00821	0.50490	0.00558	0.00363	0.980	0.950
Hydrozetidae	0.00378	0.00799	0.47310	0.00470	0.00363	0.986	0.935
Empididae	0.00331	0.00522	0.63450	0.00415	0.00343	0.991	0.963
Tanypodinae	0.00323	0.00554	0.58330	0.00531	0.00157	0.996	0.059
Lebertiidae	0.00239	0.00429	0.55700	0.00242	0.00296	1.000	0.651

Table S.9. AIC_C table for log_{10} total invertebrate abundance. Variables used in candidate models included log_{10} total suspended solids (TSS mg/L), log_{10} number of upstream slumps, and active area of the nearest slump (m^2). The global model includes the each of the listed variables. TSS was the most important variable for determining variation in abundance, followed by the number of upstream slumps (slumps), and active area (area). Year and distance to the nearest active slump were not included in AIC candidate models as they did not explain a significant amount of variation in abundance. RSS= residual sum of squares, AIC_C= Akaike Information Criterion corrected for sample size, w_i= Akaike weight.

							Importance Weight		
**Model**	**K**	**RSS**	**AICc**	**ΔAICc**	**Relative Liklihood**	**wi**	TSS	Slumps	Area
Log10 TSS, Log Upstream Slumps	3	15.39	-68.17	0.00	1.00	0.38	0.38	0.38	0
Log10 TSS	2	16.10	-67.84	0.33	0.85	0.32	0.32	0	0
Global	4	15.17	-66.70	1.47	0.48	0.18	0.18	0.18	0.18
Log10 TSS, Active Area	3	15.99	-66.00	2.17	0.34	0.13	0.13	0.00	0.13
Log Upstream Slumps, Active Area	3	19.98	-53.29	14.88	0.001	0.0002	0	0.0002	0.0002
Active Area	2	23.96	-45.18	22.99	0.00001	0.000004	0	0	0.000004
Log Upstream Slumps	2	24.03	-45.02	23.15	0.00001	0.000004	0	0.000004	0
null	1	28.80	-36.83	31.34	0.0000002	0.0000001	0	0	0
						**Sum**	1.01	0.56	0.31

Table S.10. Linear regression model selected by AIC to explain variation in total abundance across all sampling years.

Term	Estimate	Standard Error	T-value	p
Intercept	3.393	0.145	23.389	< 0.001*
Log_{10} Upstream Slumps	-0.188	0.120	-1.562	0.124
Log_{10} TSS	-0.328	0.065	-5.083	<0.001*
			**Adj R^2**	0.4307
			**P value**	<0.001*

Table S.11. AIC table for Shannon Weiner Diversity index (SWD) Variables used in candidate models included log10 number of upstream slumps, and year. The global model includes the each of the listed variables. Year was the most important variable for determining variation in SWD, followed by the number of upstream slumps (slumps). Log_{10} total suspended solids, active area, and distance to the nearest active slump were not included in AIC candidate models as they did not explain a significant amount of variation in SWD. RSS= residual sum of squares, AIC= Akaike Information Criterion, wi= weighted importance.

							Importance Weight	
**Model**	**K**	**RSS**	**AICc**	**ΔAICc**	**Relative Liklihood**	**wi**	Year	Slumps
Global	3	17.10	-62.17	0.00	1.00	0.92	0.92	0.92
Year	2	19.46	-57.04	5.13	0.08	0.07	0.07	0
Log Upstream Slumps	2	21.00	-52.69	9.48	0.01	0.01	0	0.01
null	1	24.03	-47.16	15.01	0.00	0.001	0	0
						**Sum**	0.99	0.93

Table S.12. Linear regression model selected by AIC to explain variation in Shannon-Weiner diversity (SWD) across all sampling years.

Term	Estimate	Standard Error	T-value	p
Intercept	-115.151	33.255	-3.463	0.001*
Log_{10} Upstream Slumps	0.308	0.113	2.728	<0.001*
Year	0.058	0.017	3.510	<0.001*
			**Adj R^2**	0.262
			**P value**	<0.001*

Figures

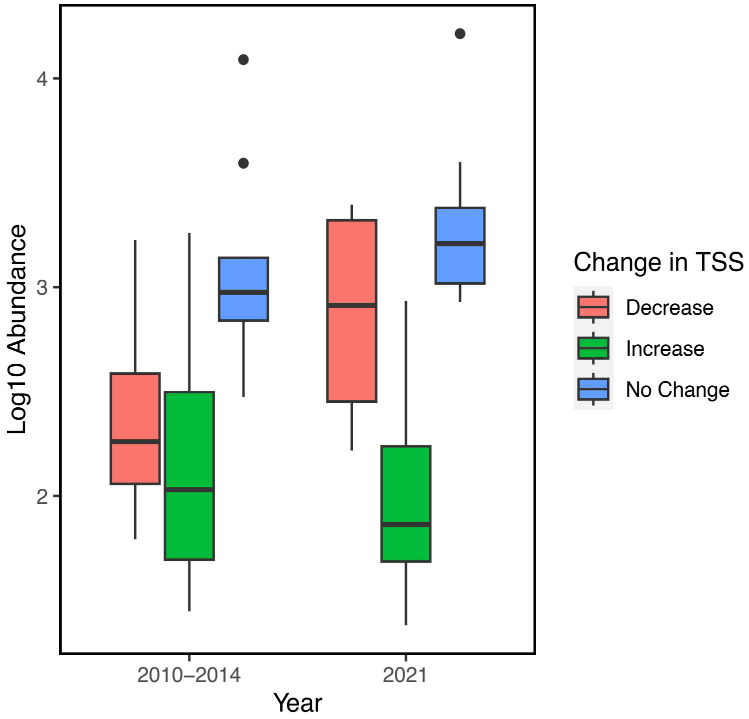

Figure S.1. Boxplot depicting abundance at sites that demonstrated change in TSS across sampling periods (see Table 1). Boxes represent the interquartile range, with the center line at the median. 'Decrease' represents sites where TSS was over 100 mg/L lower in 2021 than 2010-2014 (n=4). 'Increase' represents sites where TSS was over 100 mg/L higher in 2021 than 2010-2014 (n=11). 'No Change' represents sites that did not show large changes to TSS across sampling periods (n=9). Note that these results were not significant in the year*change interaction term in ANOVA.

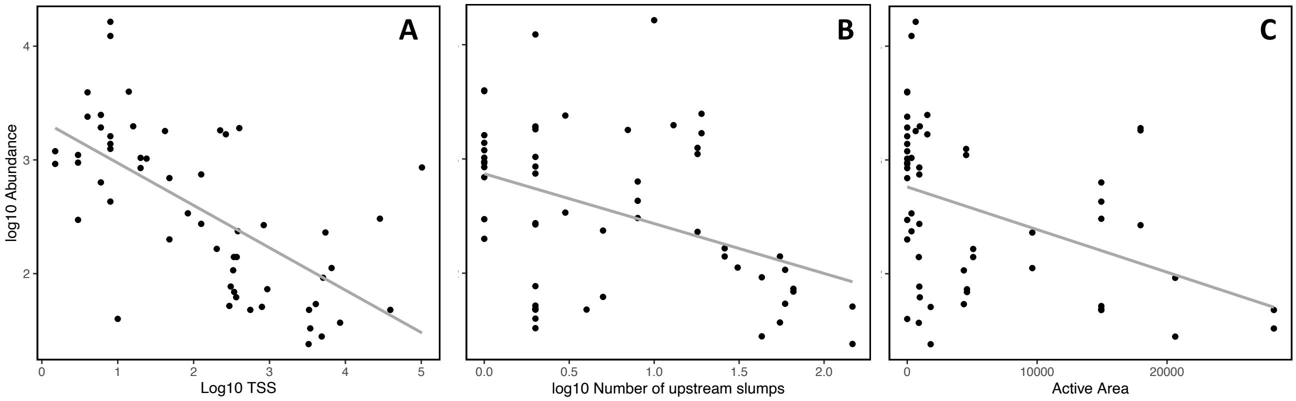

Figure S.2. Linear regressions of $\log_{10}$ Abundance with significant slump predictor variables. A) Abundance decreases as TSS increases (R^2=0.43, p=<0.001). B) Abundance decreases as the number of slumps upstream from the site increases (R^2=0.17, p=0.002). C) Abundance decreases as the active area of the nearest slump increases (R^2=0.17, p=0.002).

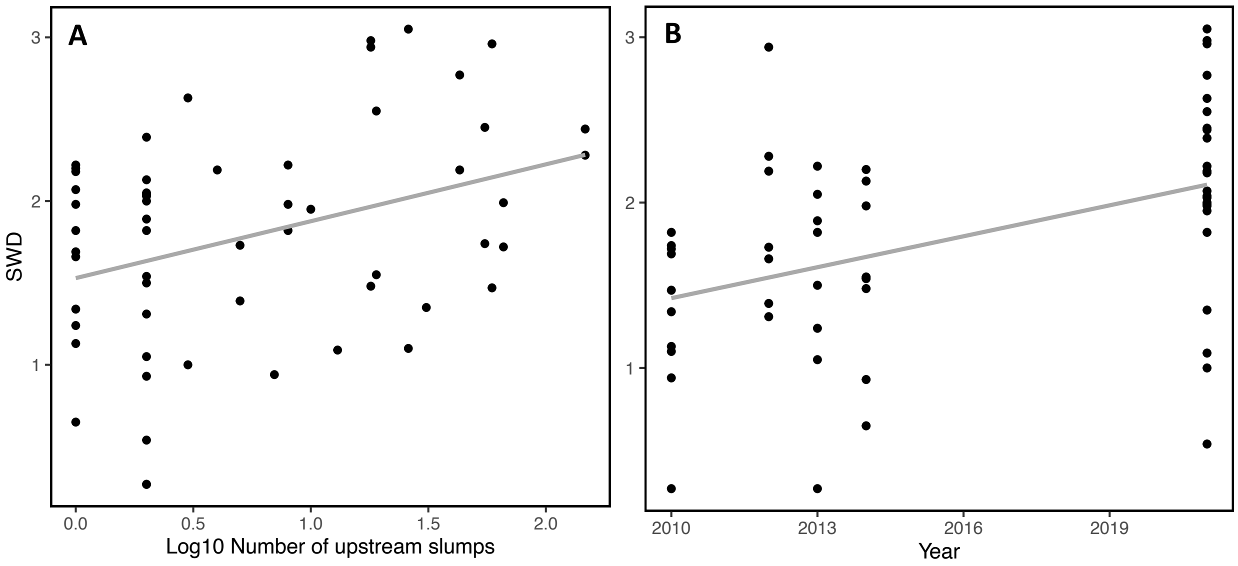

Figure S.3. Linear regressions of Shannon-Weiner diversity (SWD) with significant slump predictor variables. A) SWD increases as the number of slumps upstream from the site increases (adj R^2=0.15, p=0.002). B) SWD increases along with year (adj R^2=0.15, p=0.002).
